# Landscape-level variability and insect herbivore outbreak captured within modern forests provides a framework for interpreting the fossil record

**DOI:** 10.1038/s41598-023-36763-4

**Published:** 2023-06-15

**Authors:** Lauren Azevedo-Schmidt, Anshuman Swain, Lauren G. Shoemaker, Ellen D. Currano

**Affiliations:** 1 Climate Change Institute, University of Maine, Orono, 04469 USA; 2 Department of Botany, University of Wyoming, Laramie, 82071 USA; 3 Department of Organismic and Evolutionary Biology, Harvard University, Cambridge, 02138 USA; 4 Department of Geology and Geophysics, University of Wyoming, Laramie, 82071 USA

**Keywords:** Ecology, Palaeoecology

## Abstract

Temporal patterns of plant–insect interactions are readily observed within fossil datasets but spatial variability is harder to disentangle without comparable modern methods due to limitations in preservation. This is problematic as spatial variability influences community structure and interactions. To address this we replicated paleobotanical methods within three modern forests, creating an analogous dataset that rigorously tested inter- and intra-forest plant–insect variability. Random mixed effects models, non-metric multidimensional scaling (NMDS) ordinations, and bipartite network- and node-level metrics were used. Total damage frequency and diversity did not differ across forests but differences in functional feeding groups (FFGs) were observed across forests, correlating with plant diversity, evenness, and latitude. Overall, we found higher generalized herbivory within the temperate forests than the wet-tropical, a finding also supported by co-occurrence and network analyses at multiple spatial scales. Intra-forest analyses captured consistent damage type communities, supporting paleobotanical efforts. Bipartite networks captured the feeding outbreak of *Lymantria dispar* caterpillars; an exciting result as insect outbreaks have long been unidentifiable within fossil datasets. These results support paleobotanical assumptions about fossil insect herbivore communities, provide a comparative framework between paleobotanical and modern communities, and suggest a new analytical framework for targeting modern and fossil outbreaks of insect feeding.

## Introduction

Fossilized plant–insect interactions provide invaluable insight into true long-term patterns of temporal or regional change^1–3^, often driven by climatic^4–7^ or extinction events^8–11^. Investigating temporal change is important for understanding large-scale events, which are applicable to anthropogenic global change, but it is only part of the variability expressed within ecosystems. Disentangling spatial variability in plant–insect interactions locally within fossilized environments has been limited, but spatial variability has been shown to influence how terrestrial ecosystems respond to large-scale climatic events^12^. Fossilized plant–insect interaction data are limited by preservation and a lack of comparable modern studies, hindering our ability to understand how spatial variability is incorporated into fossil assemblages. Additionally, fossil and modern studies at present cannot be directly compared as methodologies vary in spatial, temporal, and taxonomic scale, with few exceptions^13–15^. By addressing this knowledge gap we can pose questions regarding mechanistic drivers of change using fossil ecosystems which experienced a multitude of abiotic and biotic conditions to potentially better predict future terrestrial ecosystems under global climate change.

Plant–insect interactions provide a framework for understanding temporal patterns of ecosystem collapse and rebound following devastation.A well-studied example of this within the deep-time fossil record is the Cretaceous - Paleogene extinction event (K-Pg; 66 million years ago). However, individual plant and insect herbivore communities had varying responses^9–11,16,17^, likely due to different local abiotic and biotic conditions across a spatial gradient. When spatial variability is accounted for within the fossil record, it is either across global latitudinal gradients^1^ or within specific environments which allow for high-resolution data collection. In particular, Currano (2009)^18^ and Currano et al. (2011)^19^ analyzed spatial differences in plant–insect interactions within Eocene and Oligocene paleo-forests, linking results to variability or patchiness in plant community composition. It is important to note that many Quaternary studies have worked to understand spatial variability within the context of plant–insect interactions^20–23^; however, these lack comparable methods to deep-time analyses making it challenging to compare across fossil deposits. Despite these studies, we lack knowledge regarding spatial variability within the fossil record which captures long-term (thousands to millions of years) patterns of change, providing critical information for anthropogenic climate change. The trade-off, however, is that temporal resolution is lower than in modern studies, as single fossil assemblages typically represent years to centuries. Fossil datasets account for broad-scale temporal and abiotic variables, such as climatic and extinction events, influencing plant–insect interactions, but to further understand the importance of spatial variability in structuring these communities, new modern studies are necessary.

Modern studies have focused on how landscape or habitat fragmentation, species pools, and niche space impact plant–insect interactions^24–26^; however, these relationships differ when comparing forests to one another, often across latitudinal gradients. Specifically, the pressure and intensity of herbivory, insect and plant diversity, and specialization on host-plants varies across forest types^27–32^, such as temperate vs. wet-tropical forests^13,27^. These studies highlight the importance of documenting spatial variability at regional and local scales using multiple methods, many of which are inaccessible to paleontological researchers. In order to connect across disciples we first need to investigate how and if spatial variability in plant–insect interactions is recorded, using standardized methods for measuring insect feeding.

Documenting insect herbivore communities via feeding damage types (DTs) and functional feeding groups (FFGs)^33^ on leaves is widely used within paleobotany but rarely utilized within modern ecological studies^34^. DTs are morphologically distinct patterns of insect feeding that can be statistically analyzed to quantify the frequency (the percent of leaves with any herbivore damage) and diversity (the number of DTs observed) of feeding. FFGs such as hole, margin, skeletonization, surface, piercing and sucking, mine, and gall damage encompass varying numbers of DTs which can further be categorized as generalist, often made by polyphagous insects and present on many plant species, or specialist, occurring on specific plants or related hosts^33^. DTs more closely represent individual insect herbivore species while FFGs provide a broader understanding of insect herbivore groups which possess similar mouthparts. This method accurately captures insect herbivore communities and can be used to monitor living insect communities, as recording insect herbivore communities is time-consuming and often not feasible^35^. An added benefit of standardizing modern methods to this well-established paleontological method^1^ of analyzing insect herbivory (i.e., DTs), is the creation of consistent and comparable datasets across modern and fossil records which can seamlessly be integrated^13,28,35,36^ and used to compare present-day to ancient plant–insect interactions^15^.

**Figure 1:**
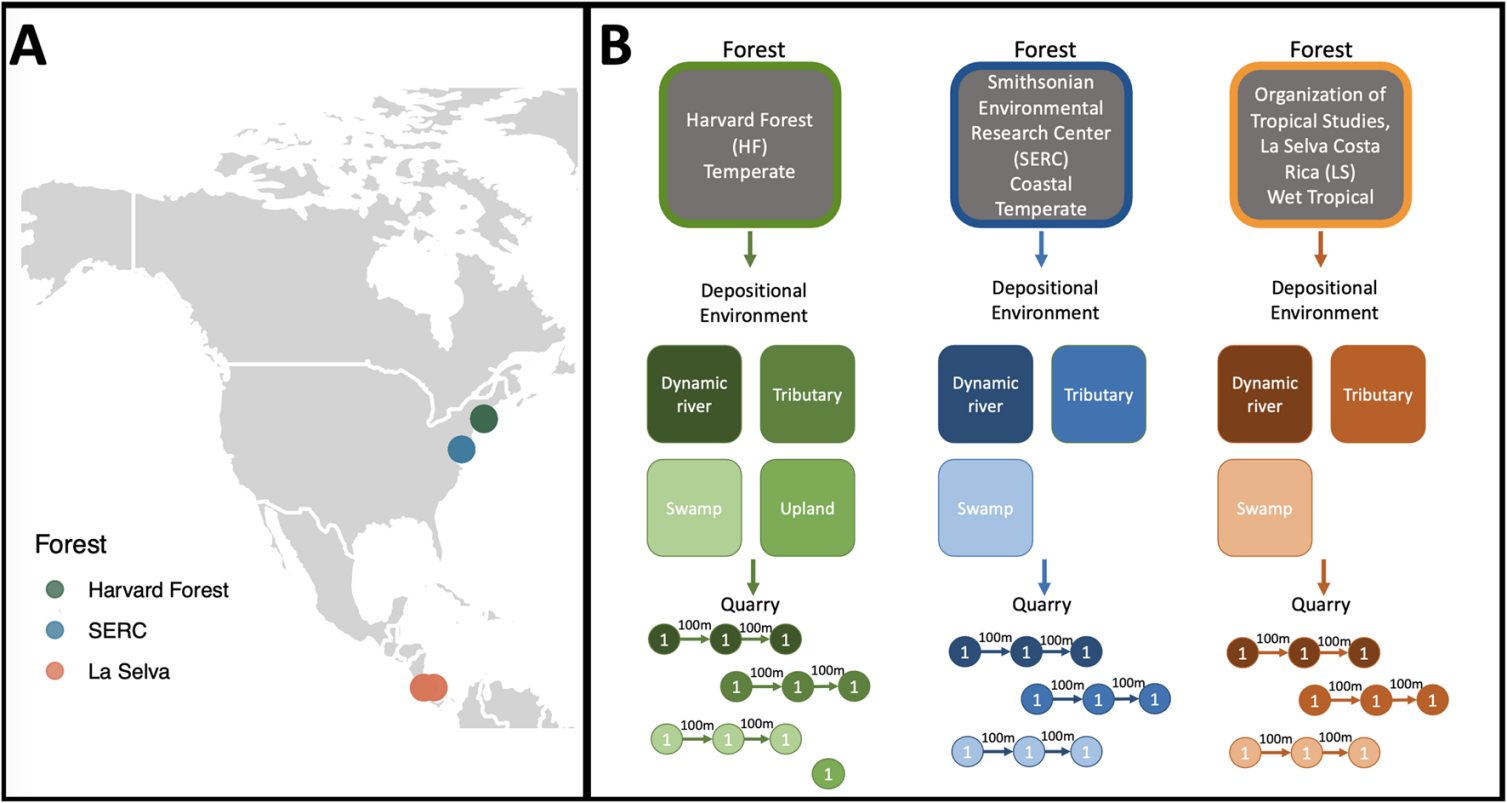
Map of sampling locations (**A**) and conceptual figure showing scales or levels of data collection (**B**). At the forest-level each ecosystem is analyzed as the mean of all data points, losing spatial variability. At the depositional environment level we increase our ability to detect variability by comparing sites within and across forest types. Below the depositional environment level is the quarry, where each point represents an individual sampling location. Each depositional environment except the upland, was sampled with three quarries, $\sim$ 100 m apart to account for spatial variability. At each quarry, $\sim$ 400 leaves were collected, totaling $\sim$ 1200 leaves per depositional environment. When we scale that to the forest-level we get 3600–4000 leaves. Colors correspond to forest type (Harvard Forest green, SERC blue, and La Selva orange), with color gradient differentiating depositional environments.

**Figure 2:**
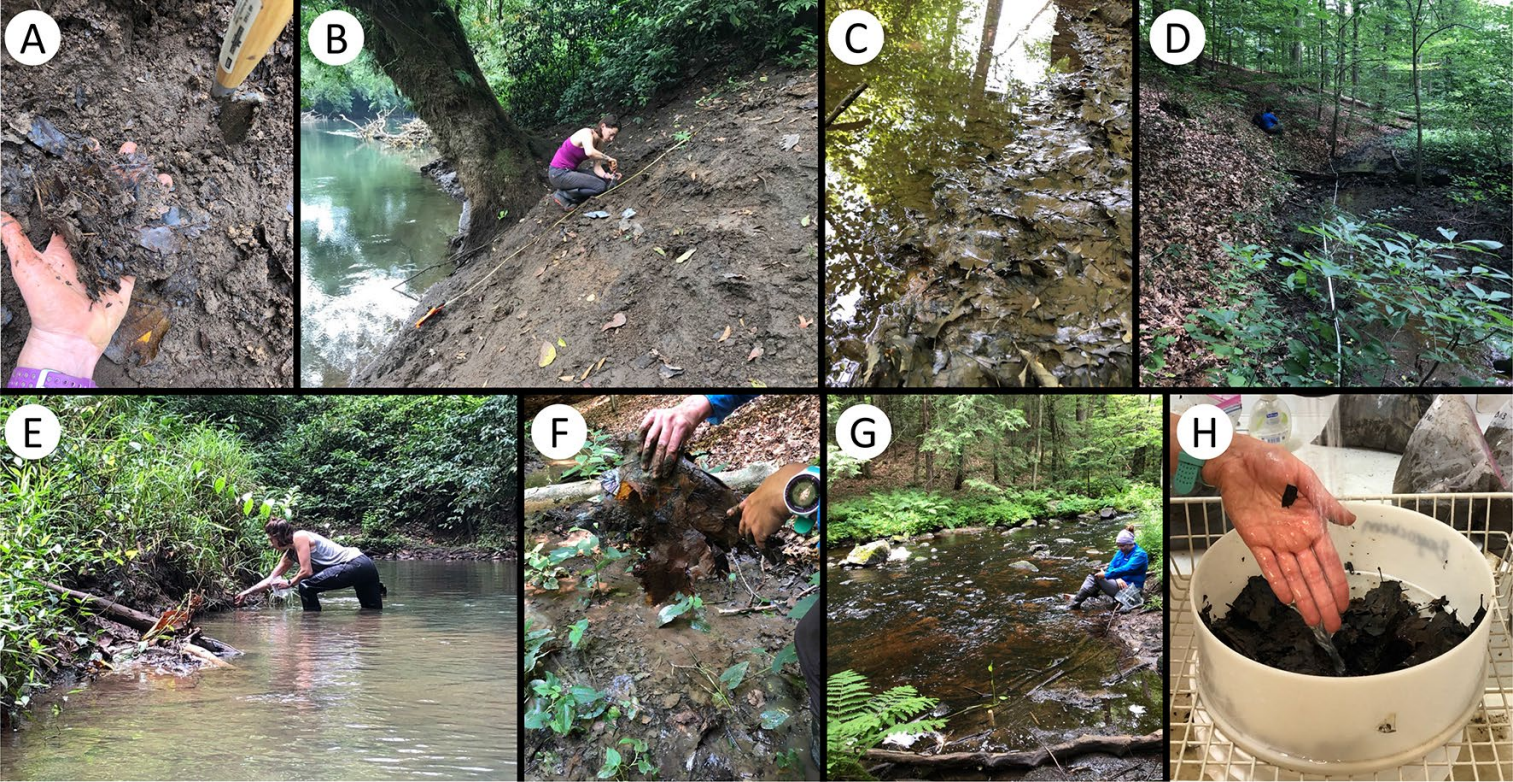
Plate showing sampling methods across all forest types. Examples of what leaves look like coming out of the sediment, trowel for scale (**A**), and sampling within the dynamic river (**B**) and tributary (**E**) at the La Selva Biological Research Station. Dense leaf packs (**C**,**F**) and overbank sampling (**D**) at the Smithsonian Environmental Research Center (SERC). Sampling from within fine sediment at Harvard Forest (**G**). Leaves were carefully rinsed of sediment prior to drying and pressing (**H**).

**Figure 3:**
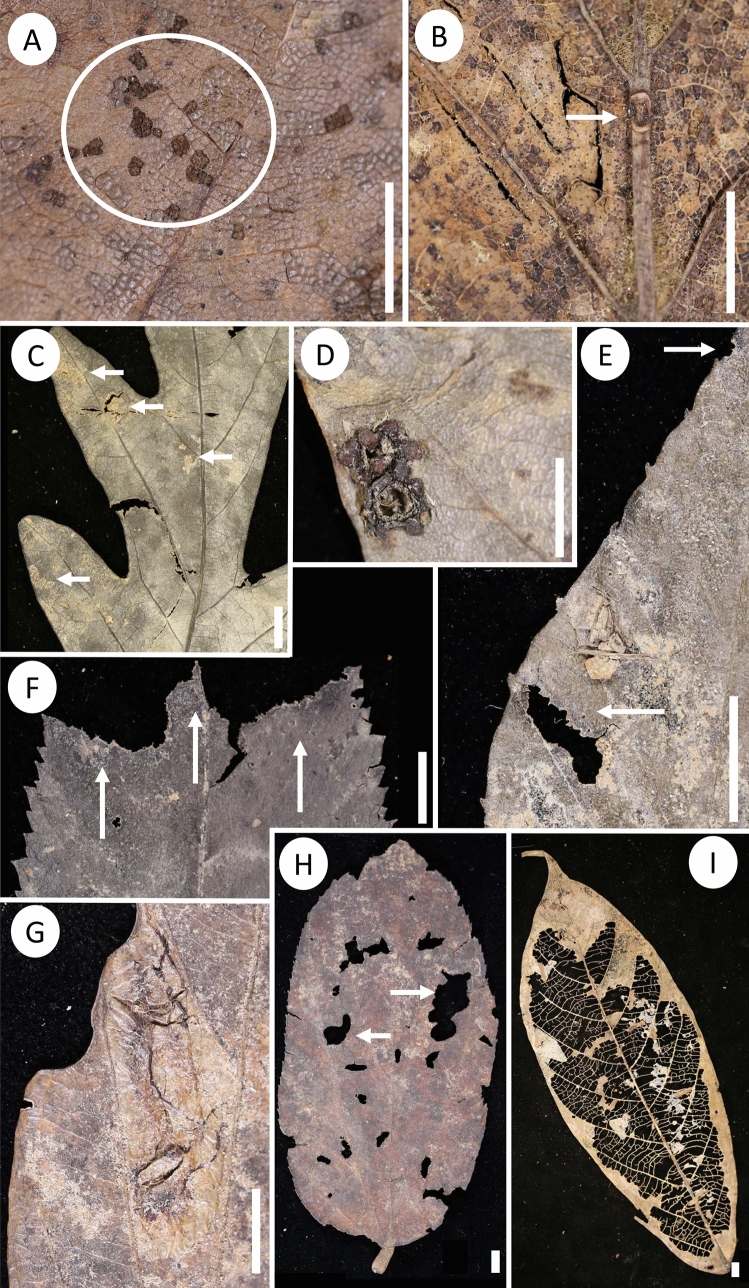
Examples of DT across all three forests. Three new DTs were found for this study, DTS1 (**A**; surface feeding;HF1901.1 #273), DTN (**B**; surface feeding;HF1903.3 #369), and DTG5 (**D**; gall damage; MD1903.2 #161). DT333 (**C**; surface feeding) is very common at Harvard Forest shown here on a white oak (MD1901.1 #18). DT3 (**E**; lower arrow; hole feeding) and DT12 (**E**; upper arrow; margin feeding) on an American beech (*Fagus grandifolia*) leaf (MD1902.2 #79), are common general damage patterns along with DT13 on an American hornbeam (*Carpinus caroliniana*) leaf (**F**; margin feeding; MD1902.2 #154). Specialized damage such as DT37 (**G**; mine damage) found on *Zygia longifolia * (LS1901.3 #110), generalized damage such as DT5 (**H**; hole feeding) on *Luehea seemannii* (LS1901.1 #254), and DT16 (**I**; skeletonization) on *Trophis racemosa* (LS1903.3 #195) are all found within La Selva. Scale bars are all 0.50 cm.

Understanding spatial variability in plant–insect interactions at the inter- and intra-forest level, specifically to bridge modern and fossil datasets, allows for more accurate predictions and drivers of change. To achieve this, we present novel methods of analyzing spatial variability in plant–insect interactions using paleontological methods of assessing DTs and FFGs on fallen leaves within three modern forests: temperate Harvard Forest (HF), coastal temperate Smithsonian Environmental Research Center (SERC), and wet-tropical La Selva (LS; Figs. 1, 2, and 3). To replicate paleobotanical studies, leaves were excavated from within the sediment across readily preserved depositional environments (i.e. rivers, streams, swamps) and similar questions were asked. Additionally, we sought to rigorously investigate how inter- and intra-forest patterns of herbivory differed and if the known insect outbreak of *Lymantria dispar* at HF could be detected. Specifically we asked, (1) is total insect herbivory frequency and diversity greater within tropical LS? (2) do FFG frequencies considerably differ across HF, SERC, and LS? (3) does analyzing DTs capture inter- and intra-forest variability, specifically landscape heterogeneity? and (4) is the outbreak of *L. dispar* captured within the data? These specific questions align our modern study with fossil studies, bridging the gap between the two disciplines and providing a better understanding of past, present, and future relationships between plant and insect herbivores.

## Results

**Figure 4:**
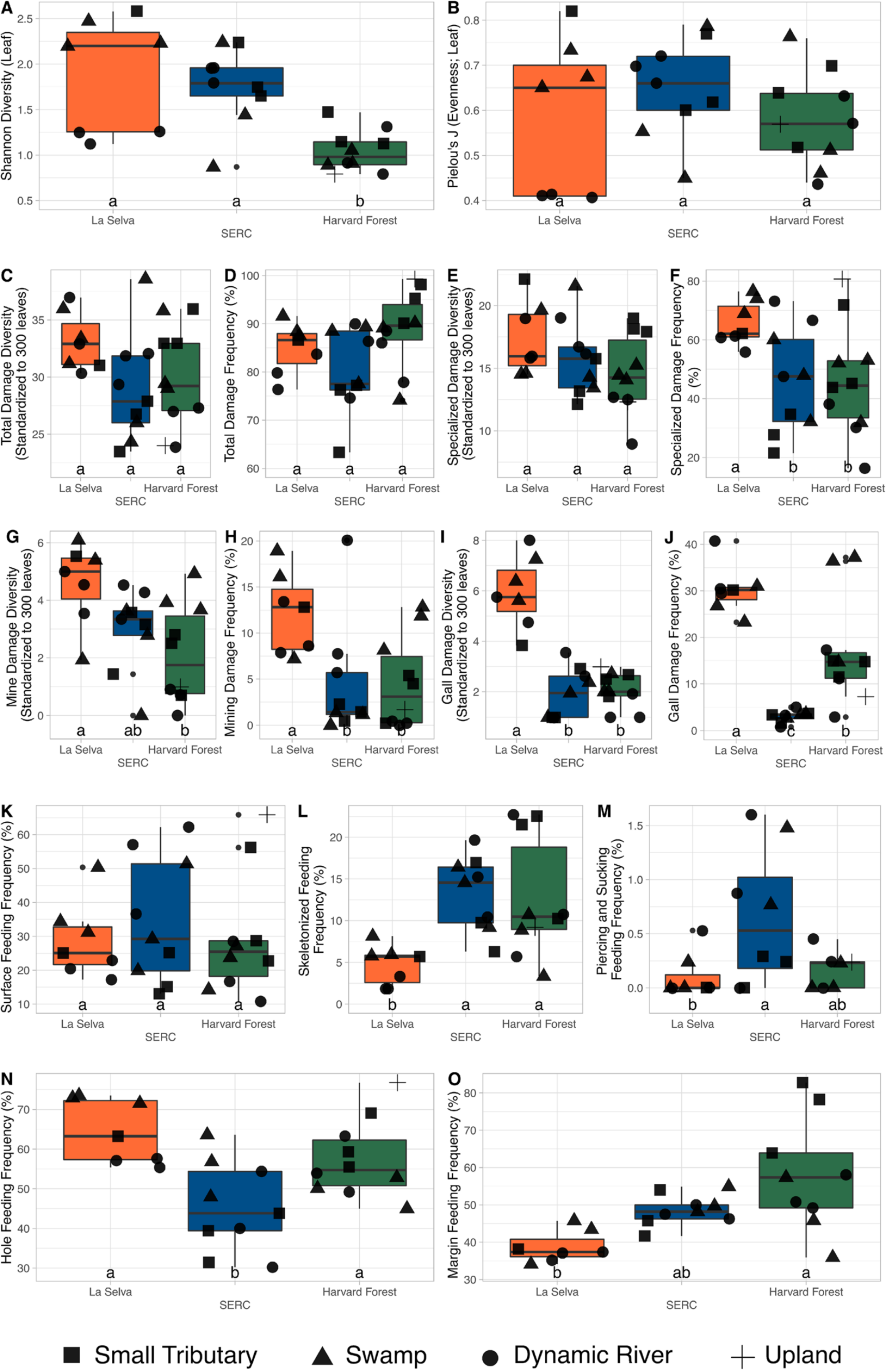
Forest-level variability in plant diversity (**A**) and evenness (**B**), and insect herbivory (**C**–**O**) for La Selva (orange), SERC (blue), and Harvard Forest (green). Multiple quarries are represented by points which correspond to the depositional environment (square = small tributary, triangle = swamp, circle = dynamic river, and plus = upland). Boxplots show the minimum and maximum values (whiskers) along with the first quantile (Q1), mean (Q2), and third quantile (Q3; boxes). DT diversity metrics were standardized to 300 leaves to account for uneven sampling bias.

HF had significantly lower plant diversity than LS (p $\le$ 0.01) or SERC (p $\le$ 0.01), but plant evenness did not differ across forests (p = 0.57, 0.99, and 0.45; Fig. 4A,B). Total damage diversity and frequency did not differ across forest type (p = 0.13, 0.26, 0.88 and p = 0.49, 0.55, 0.06; Fig. 4C,D). Specialized damage diversity was consistent across forests (p = 0.57, 0.16, 0.64; Fig. 4E), but specialized damage frequency was highest at LS (p = 0.06; Fig. 4F; Table S1). Mine diversity (specialized FFG) is greater within LS than HF (p $\le$ 0.01) but similar to SERC (p = 0.12; Fig. 4G), and mine frequency (specialized FFG) at LS is greater than SERC (p = 0.02) and HF (p = 0.02), with no significant difference between the two temperate forests (p = 0.99; Fig. 4H). Diversity and frequency of gall damage (specialized FFGs) within LS was significantly greater, compared to HF (p $\le$ 0.01) and SERC (p $\le$ 0.01; Fig. 4I,J). Surface feeding (generalized and specialized FFG) does not differ between forest types (p = 0.78, 0.99, 0.79; Fig. 4K), but skeletonized damage frequency differs between LS and SERC (p = 0.01) and HF (p = 0.012), with the highest values in the temperate (Fig. 4L). Piercing and sucking damage is only different between SERC and LS (p = 0.04); however, piercing and sucking is highly biased by preservation and likely underestimated at all sites (Fig. 4M). Interestingly, hole feeding frequency (which can range from generalist–specialist FFG) is not significantly different between HF and LS (p = 0.35) but is significantly lower at SERC than at LS (p $\le$ 0.01) or HF (p = 0.04; Fig. 4N). Lastly, margin feeding (generalist FFG) is highest at HF and lowest at LS (p $\le$ 0.01; Fig. 4O). See Supplementary Table S1 for all p-values.

Depositional environment influences intra-forest patterns on plant diversity and evenness (Fig. 4A,B) along with patterns in individual FFGs (Fig. 4C–O). We found the greatest variability in plant diversity and evenness within LS, with consistently low diversity at the dynamic river sites (circles) and higher diversity at the small tributary (squares) and swamp (triangles) sites (Fig. 4A,B), a result not shared by the other forests. Both temperate forests have great variability in plant diversity and evenness within swamp depositional environments, with points plotting tighter together for dynamic river and tributaries (Fig. 4A,B). This is most apparent for plant evenness within dynamic river (circles) environments within the SERC landscape (Fig. 4B). Multiple other herbivory metrics appear to be influenced by depositional environment, notably frequencies of total damage, specialized damage, surface feeding, skeletonization, and hole feeding (Fig. 4D,F,K,L,N). Dynamic river environments have low values relative to other depositional settings.

The influence of biotic factors (plant community diversity and evenness) versus cross-forest abiotic differences (represented as latitude) on DT frequencies and diversities was assessed using mixed effects models. An increase in latitude increased skeletonization frequency (estimate = 0.53, p = 0.01; Table S2) while negatively influencing gall diversity (estimate = − 1.98, p $\le$ 0.01; Table S3). As plant diversity increases, mine damage diversity increases (estimate = 1.13, p = 0.07), though this relationship was only marginally significant (Table S3). All other herbivory metrics showed no strong or significant relationships with plant diversity, evenness, nor latitude.

**Figure 5:**
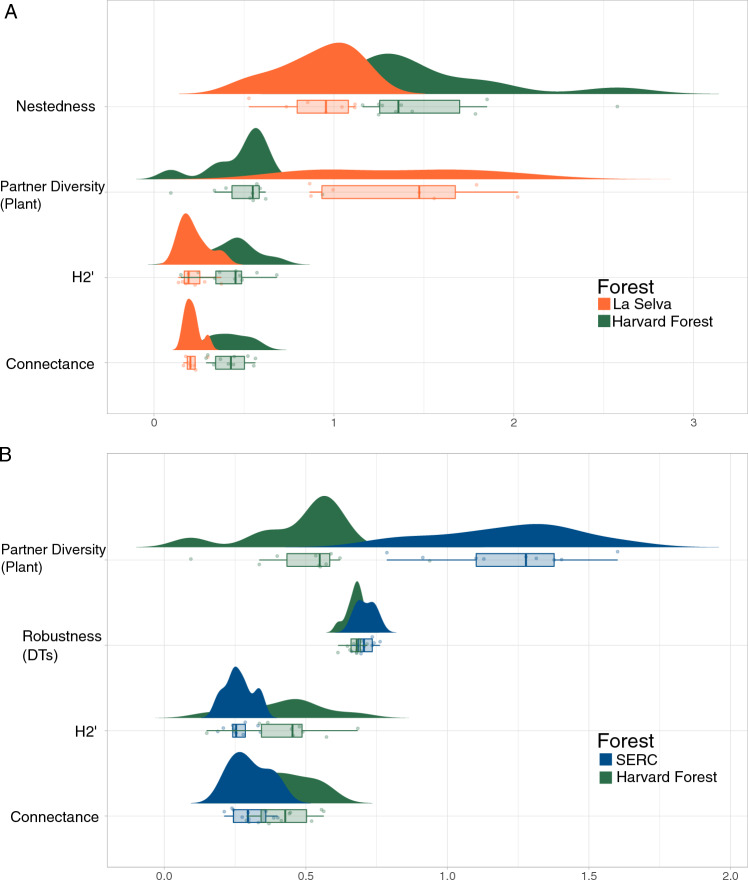
Bipartite analysis at the network-level show differences across forest types. Harvard Forest and La Selva have the greatest network property differences (**A**) with differences also occurring across SERC and Harvard Forest (**B**). Each point in the plot represents the network-level (quarry-level) values for a given property. The shaded areas represent density plots with the peaks hovering over the mean values. Box-plots shown below density plots show the spread of the data and raw data points with the lower and upper limits (Q1 and Q3) along with the mean values (Q2). Nestedness, partner diversity (plants), H2’, and connectance were all significantly different when comparing Harvard Forest and La Selva (**A**), while partner diversity (plants), robustness (DTs), H2’, and connectance were significantly different for Harvard Forest and SERC (**B**).

Bipartite analyses at the network-level, also referred to as forest-level (Figs. 1, 5), show significant differences in nestedness (structure of interactions), partner diversity (plant; diversity of DTs on plant taxon), robustness (DTs; effect of plant extinctions on DTs), H2’ (specialization), and connectance (interactions realized). Differences in these network metrics were found between HF and LS (excluding robustness; Fig. 5A), and HF and SERC (excluding nestedness; Fig. 5B). Nestedness, a measure of overlapping interactions, within HF is greater than LS, indicating more overlapping interactions of plants and DTs, and also includes a much larger range of values (p $\le$ 0.01; Fig. 5A). LS has greater partner diversity (i.e., diversity of DTs on a plant taxa) than HF, indicating higher host-plant diversity (p $\le$ 0.01; Fig. 5A); this metric is also higher for SERC than HF (p $\le$ 0.01; Fig. 5B). Robustness of DTs is greater within SERC than HF (p = 0.04; Fig. 5C), suggesting higher resistance of DTs to secondary extinctions from primary random removal of plants and indicating a more consistent assemblage. An interesting and unexpected result, however, is that H2’ is greater within HF than either LS or SERC (p $\le$ 0.01 and p $\le$ 0.01; Fig. 5A,B). All other bipartite network metrics indicate that HF is less specialized than LS and SERC; however, H2’ shows the opposite results. Although H2’ is often used to characterize specialization, here it characterizes insect feeding preference during an outbreak because we compared multiple bipartite network metrics, rather than relying on a single metric of specialization. Connectance is greater within HF than LS or SERC (p $\le$ 0.01 and p $\le$ 0.01; Fig. 5A,B), as is nestedness (p $\le$ 0.01; Fig. 5A), supporting other network metrics which found HF to have more generalized interactions than LS or SERC. Lastly, there were no forest-level differences between SERC and LS, likely due to low statistical power. See Supplementary Table S4 for complete bipartite network metric outputs.

**Figure 6:**
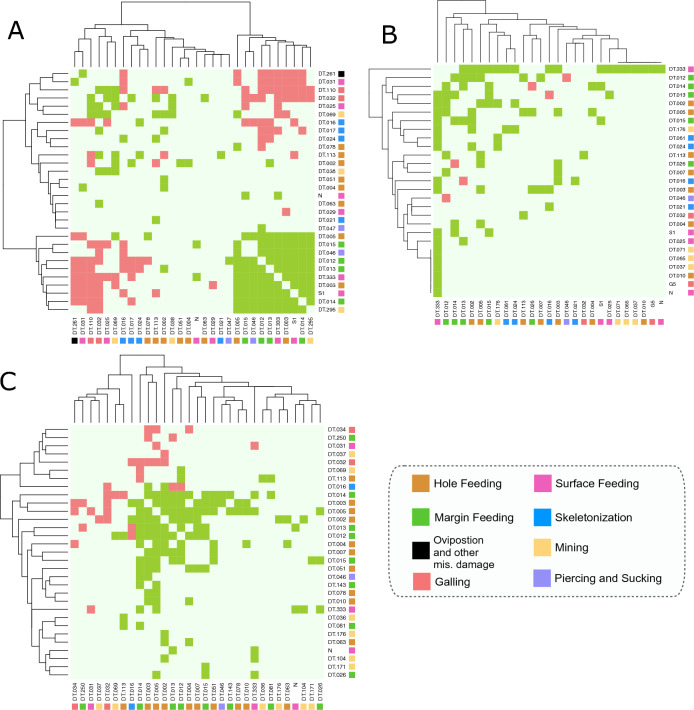
Co-occurrence and pairwise analysis for Harvard Forest (**A**), SERC (**B**), and La Selva (**C**) showing a strong positive (green) and negative (pink) co-occurring DTs. Mint green background color corresponds to random chance and thus, neither a positive or negative relationship occurs.

Co-occurrence analyses show distinct groups of positively and negatively co-occurring DTs within each forest. The strongest co-occurrence relationships appear within HF and LS (Fig. 6A,C and S1, S3). HF DTs have both strong positive and negative co-occurrence pairings. DT 5 and 3 (large and medium polylobate hole feeding; Fig. 3E,H), 12–15 (various margin feeding morphologies; Fig. 3E,F), 46 (circular punctures $\le$ 2 mm diameter, central depression; piercing and sucking), S1 and 333 (morphologically distinct feeding of leaf surface tissue; surface feeding; Fig. 3A,D) and 295 (distinct “cavities” created by a developing larva; mining) tend to occur on the same leaves (Fig. 6A). Additionally, these DTs strongly negatively co-occur with DT 261 (thick circular exterior rim with detached dark central ovoidal marking; piercing and sucking), 31 (removal or abrasion of surface tissues with a distinct circular to ellipsoidal reaction rim; surface feeding), and 110 (large, on 3rd order veins, ovoidal-circular; central chamber sharply separated from thick carbonized brim; gall). SERC has fewer co-occurrences than other forests but DT333 co-occurs with several DTs, such as DT N (new surface feeding), DT G5 (new gall; Fig. 3D), DT 10 (excised tissue ring with loosely attached central disc; hole feeding), DTs 37, 65, and 71 (mining), and DT 25 and S1 (surface feeding; example of S1 Fig. 3A). Interestingly, negative relationships are sparse at SERC (Figs. 6B and S2). LS has strong positive co-occurrence relationships among hole feeding DTs 2, 3, and 5 (various shaped and sized holes) and margin feeding DTs 12 - 14. DT16 (skeletonization with a poorly developed reaction ring; Fig. 3I) strongly negatively occurs with DTs 12 and 13. Other strong negative relationships occur between DT32 (non-descript galls occurring on tissues between major veins) and the other positively occurring DTs (Fig. 6C).

**Figure 7:**
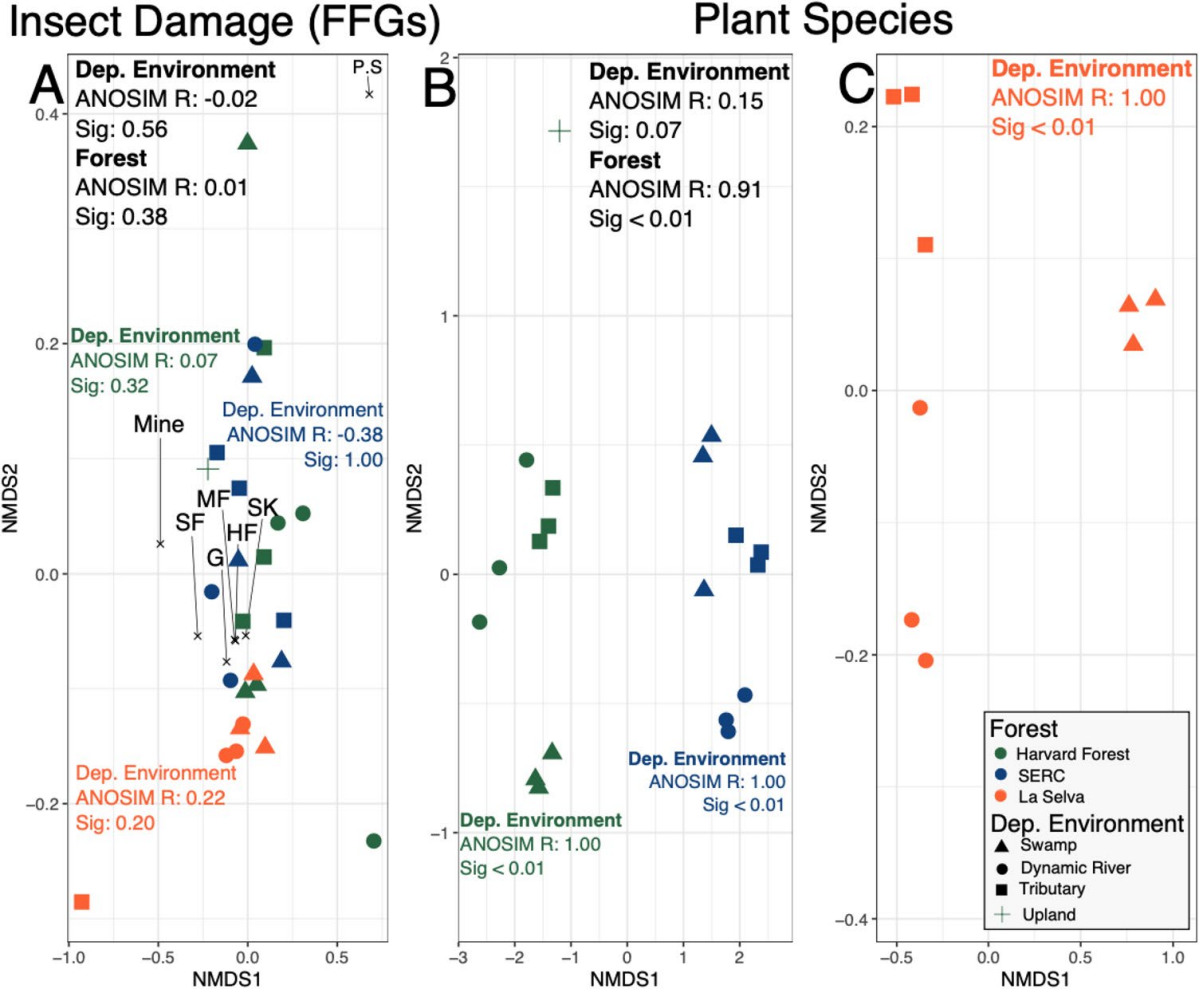
NMDS plots showing inter- and intra-forest variability in FFGs (**A**) and plant communities (**B**,**C**) for Harvard Forest (green), SERC (blue), and La Selva (orange). Shapes correspond to depositional environment, with multiple quarries within each depositional environment, except for the one upland (+) location at HF. Two quarries within the tributary (square) depositional environment of La Selva did not have enough leaves ($\ge$300) and were removed from the analyses. FFG abbreviations are as follows: hole feeding (HF), margin feeding (MF), skeletonization (SK), gall (G), mine (M), surface feeding (SF), and piercing and sucking (P.S). Plant communities were directly compared between Harvard Forest and SERC (**B**) with La Selva analyzed by itself due to a lack of shared plant species (**C**). ANOSIM analyses were conducted for each NMDS plot. Inter-forest variability was examined for FFGs (**A**) and plants (**B**) using forest and depositional environment as the a priori groups (black text) while intra-forest variability of FFGs (**A**) and plants (**B**,**C**) was examined using only depositional environment as the a priori group (colored text). La Selva plant species were only examined at the intra-forest scale (**C**).

FFG distributions and plant species composition show different degrees of spatial changes across and within forests (Fig. 7). Differences in FFG distributions (i.e., the percent of leaves in a sample with each FFG) across forests (ANOSIM R = 0.01) and depositional environments (R = − 0.02) are minimal (Fig. 7A). This pattern of non-significant differences in FFG distributions is also shared within each forest when comparing depositional environments (R = 0.07 for HF; − 0.38 for SERC, and 0.22 for LS; Fig. 7A). In contrast, plant species composition differs at both the inter- and intra-forest scale (Fig. 7B,C). At the inter-forest level, LS has no plant species in common with the two temperate forests, and plant communities within HF are more similar to each other than to plant communities within SERC (R = 0.91, sig. $\le$ 0.01; Fig. 7B). Plant species composition within each depositional environment for HF (swamp, dynamic river, tributary, and upland) are more similar to each other than the overall forest community (R = 1.00, sig. $\le$ 0.01). This pattern also occurs at SERC (Fig. 7B; R = 1.00, sig. $\le$ 0.01) and LS (R = 1.00, sig. $\le$ 0.01; Fig. 7C). Depositional environment for all forests is thus a strong driver of plant communities, and this spatial variability at the intra-forest scale is preserved within the data.

**Figure 8:**
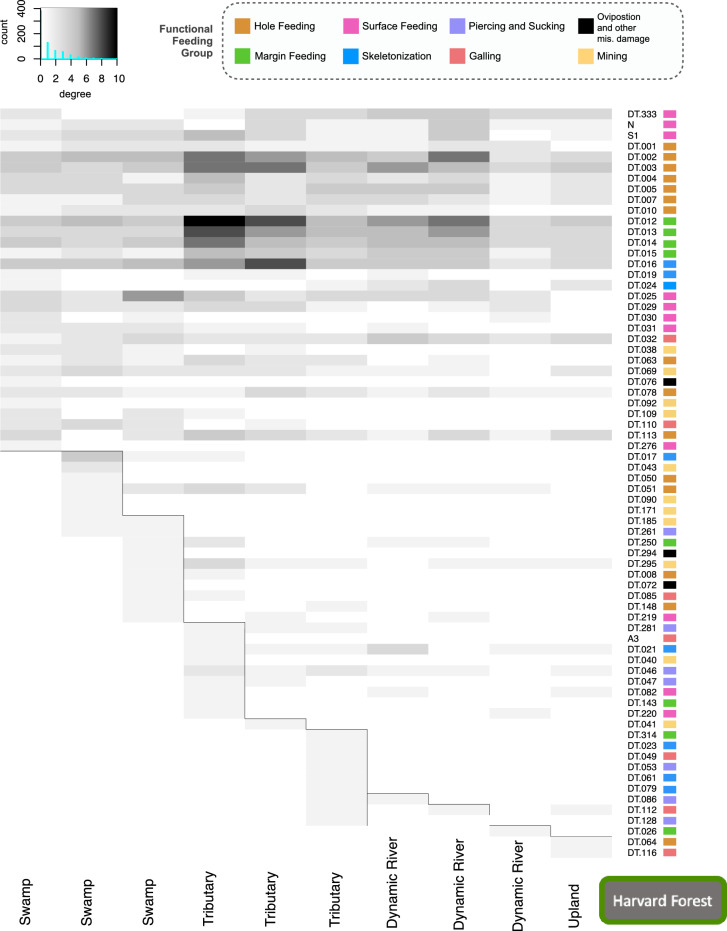
Bipartite analysis of degree, at the node or DT-level (for each quarry) shows the importance of microhabitats within Harvard Forest. Darker colors represent high generalization, interactions of DTs with many plants, and lighter colors signify specialization, interaction of DT with few plants. White indicated no occurrence. Grey lines were added to highlight the specific DT groupings across quarries. Histograms at top left corner shows the distribution of occurrence with degree on the x-axis showing zero to ten, with specialization occurrence increasing with degree, while the y-axis is count or occurrence of DTs. FFGs are color coded to the right of each DT with the legend shown at the top.

**Figure 9:**
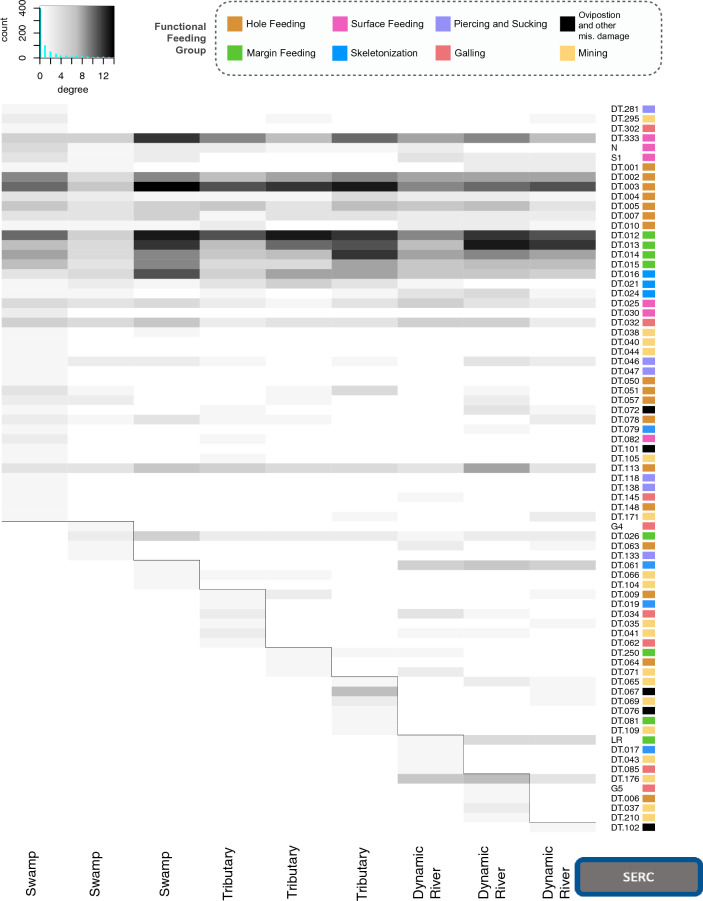
Bipartite analysis of degree, at the node or DT-level (for each quarry) shows the importance of microhabitats within SERC. Darker colors represent high generalization, interactions of DTs with many plants, and lighter colors signify specialization, interaction of DT with few plants. Grey lines were added to highlight the specific DT groupings across quarries.Histograms at top left corner shows the distribution of occurrence with degree on the x-axis showing zero to ten, with specialization occurrence increasing with degree, while the y-axis is count or occurrence of DTs. FFGs are color coded to the right of each DT with the legend shown at the top.

**Figure 10:**
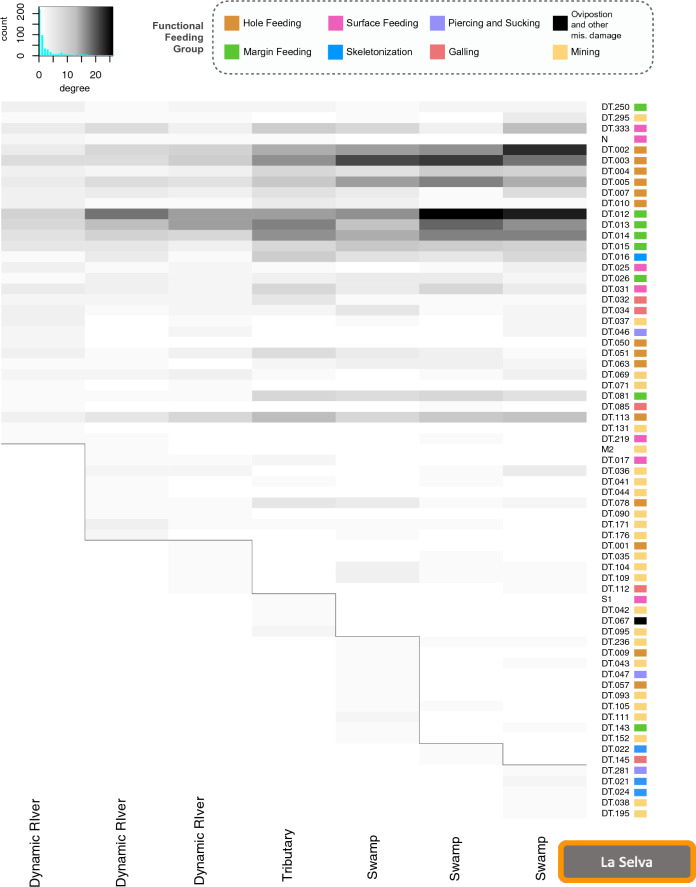
Bipartite analysis of degree, at the node or DT-level (for each quarry) shows the importance of microhabitats within La Selva. Darker colors represent high generalization, interactions of DTs with many plants, and lighter colors signify specialization, interaction of DT with few plants. Grey lines were added to highlight the specific DT groupings across quarries. Histograms at top left corner shows the distribution of occurrence with degree on the x-axis showing zero to ten, with specialization occurrence increasing with degree, while the y-axis is count or occurrence of DTs. FFGs are color coded to the right of each DT with the legend shown at the top.

Quarry-level (Fig. 1), network analyses for the degree (DT), the number of plants on which a DT occurs, (Figs. 8, 9 and 10), show heterogeneous groups of DTs within quarries at all three forests. This metric for measuring general vs. specialized insect damage differs from the standard definition^33^ but rather, looks at the occurrence of DTs across a landscape. Degree for a given DT is whether it occurs on many plant species (i.e., general; dark shades in Figs. 8, 9 and 10; dark color), or occurs on fewer species (i.e., specialized; light shades in Figs. 8, 9 and 10). True white means there are zero occurrences. All three forests show similar occurrence patterns with greater occurrences of general DTs (top portion of figures) and distinct groupings of DTs within individual quarries that are not shared with the closest sampling location. Simply stated, each quarry has distinct DT “communities” that are not shared. For example, HF has many shared DTs occurring at all quarries with varying strengths of degree; DT12 (non-distinctive cuspate margin feeds) is very general (black) within the first (left to right) tributary quarry but slightly less general at the last tributary quarry (Fig. 8). DT12 is shared across all quarries unlike DT116 (small, columnar leaf galls) which only occurs on leaves at the upland location (Fig. 8). The highly general DTs defined by Labandeira et al. (2007)^33^ tend to also have a high degree of occurrence generality on many leaves within all forests. Within the three forests examined here, we find that DT2 and DT3 (hole feeding FFG), DT12–DT15 (margin feeding FFG), and DT16 (skeletonization FFG) have a high degree of occurrence; this is an expected result as these DTs can be made by many insect species. Additionally, blocks of DTs occurring at only one, or minimally shared, quarry and interacting with few leaves (specialization) are seen within each quarry (Figs. 8, 9 and 10), suggesting high spatial variability even at this fine of a scale. Interestingly, one HF tributary quarry shares DT86 (outer rim surrounds internal lobed area; piercing and sucking FFG) with a single dynamic river quarry, and DT112 (rimmed margin with sinuses, faintly concentric and avoids primary vein; gall FFG) with another one (Fig. 8). Analyses were run with and without singletons, DTs which only occur on one leaf, and the patterns discussed above did not change (Figs. S4–S6).

## Discussion

The power of analyzing plant–insect interactions across varying spatial scales using insect herbivory (i.e., damage types and functional feeding groups) is shown here. To complement paleobotanical studies, we quantified differences in total damage frequency and diversity, as well as FFGs for two temperate forests, Harvard Forest and the Smithsonian Environmental Research Center, and one wet-tropical forest, La Selva. We found total damage frequency and diversity to be equivalent across all three forests, while FFGs differ with higher specialization observed within LS (questions 1 and 2; Fig. 4). Inter- and intra-forest heterogeneity in herbivory was recorded using the DT system and a variety of quantitative methods (question 3; Figs. 6, 7, 8, 9 and 10 and S1–S3). Although we find variation within a landscape, the distribution of FFGs are consistent supporting paleontological efforts in capturing insect herbivore communities. Lastly, bipartite networks captured feeding preference of *Lymantria dispar* and documented its known outbreak at HF (question 4; Fig. 5). The patterns we discovered in our investigation of questions 1–3 are driven by numerous abiotic and biotic mechanisms which will be expanded on; however, as we did not directly test mechanistic drivers, these remain hypotheses. Factors such as plant traits were not investigated within the study, but are the subject of forthcoming work.

Total damage frequency and diversity was similar across all forests when investigating broadly (Fig. 4C,D). This is surprising as the biotic driver, higher biodiversity within the tropics^37,38^, is assumed to increase insect herbivory^27^. Another biotic mechanism for greater insect herbivory within the tropics is leaf lifespan. Evergreen growth habits exist in both temperate and wet-tropical forests, but the vast majority of broad-leaved angiosperms in temperate forests have short leaf lifespans and are deciduous^39^, whereas broad-leaved angiosperms in wet-tropical forests tend to have longer leaf lifespans and are commonly evergreen^40,41^. Thus, we might expect insect herbivory on individual leaves to be greater within wet-tropical forests like LS, which would scale up to higher damage diversities and frequencies than observed at HF or SERC. This expectation is also connected to an abiotic mechanism: growing season length. The growing season is continuous in wet-tropical forests, whereas in cool-temperate forests, such as HF, it is constrained by frost and freeze. This limits cool-temperate insect herbivore communities to only feeding during part of the year and suggests that temperate insect herbivores feed at such an elevated rate during their restricted growing season that they match consumption by the wet-tropical insects that can feed nearly year-round. Adams et al. (2009)^28^ found that when growing season length was accounted for, temperate forests experienced greater insect herbivory than tropical forests. Similarly, a meta-analysis by Moles et al. (2011)^31^ found few differences between herbivory at high vs. low latitudes.

Differences in observed FFGs across forests (Fig. 4) is likely due to variability in insect species pool, which was previously recorded within the living community^42^. Published census data for HF and LS insect communities show differences in insect species pools and higher species diversity within LS^42^. Higher diversities of Coleoptera, Hemiptera, and Diptera were observed at LS compared to HF, whereas Hymenoptera diversity was significantly higher at HF^42^. This result is likely driven by an abundance of Ichneumonidae within HF. The variability of these groups between the two forests can be directly applied to the FFG results shown here. Specifically, leaf mine damage is significantly greater within LS than HF (Fig. 4G,H), a pattern likely driven by a high diversity of Coleoptera and Diptera^43^; we note, though, that Lepidoptera were omitted from the Archibald et al. (2016)^42^ study. Additionally, insects that gall are abundant within the Diptera and Hemiptera^44^, both dominant orders at LS, possibly driving the higher frequency and diversity of gall damage (Fig. 4I,J). Although not all species within these orders are herbivorous, they provide a broad understanding of the patterns observed within the FFG record presented here. As discussed above, variation in plant communities should influence insect herbivory and feeding, but although we have substantial differences in plant communities across forests (Figs. 4A,B and 7B,C), there is considerable overlap in FFGs (Fig. 7A).

Herbivory is intimately linked to host-plants, specifically leaf tissue, providing information about how insect herbivores interact with their food source but also with each other. Positively co-occurring DTs could provide information regarding coexistence or life stage^45^, while negatively co-occurring DTs could show competition. Competition for leaf materials influences plant–insect and insect-insect interactions; however, the landscape itself also influences these interactions. All three forests are fragmented via human infrastructure (roads, houses, etc.) and water ways (river, streams, swamps, etc.), which has previously been shown to increase insect herbivore diversity^46^. Each forest is unique in how it is fragmented, and this may cause co-occurrences to vary (Fig. 6). Interestingly, the forest that should experience greater fragmentation pressures due to forest ecosystems abruptly ending at fresh and brackish waters, SERC, generally had weak co-occurring DTs (Fig. 6B). However, a few DTs displayed strong interactions with each other, including DT333 (surface feeding FFG) positively co-occurring 100% of the time with other DTs (n = 17) and DT46 (piercing % sucking FFG) negatively co-occurring 100% of the time (Fig. S2). Conversely, HF and LS have strong co-occurrence of DTs (Figs. 6A,C; S1 and S3). HF has ten positively co-occurring DTs (Fig. S1), while only one DT, DT29 (surface feeding), negatively occurs 100% of the time. LS has five DTs which negatively occur 100% of the time, possibly indicating higher competition or more finely partitioned niche space (Fig. 6C and S3). Competition for leaf material can be mechanistically explained, as certain types of DTs remove higher amounts of leaf tissue, a precious resource. For example, skeletonizing insects remove leaf lamina, leaving behind only vein material which is less nutritious^47^, prompting other insects to avoid the leaf.

Given these observed patterns in herbivory across the three forests, it is critical to also investigate plant–insect interactions at a different scale, the intra-forest scale. Spatial variability within forests can greatly influence species distributions and interactions, and may obscure differences among forests. Patterns and changes in landscape heterogeneity, often referred to as a “mosaic”, within a forest are well-studied within the modern literature^48,49^. These pockets of interactions often create micro-habitats for the communities occupying them^50^, an important feature of the overall landscape function and an idea heavily supported by our research (Figs. 6, 7, 8, 9 and 10). Variables influencing micro-habitats include abiotic variables such as micro-climate, soil type, and nutrients alter local species composition^51^. When considering paleontological data, preservational bias must also be considered^52,53^. Depositional environment influences fossil assemblages (Fig. 7B,C) not just because different plant and insect species occur in these different micro-habitats, but also because leaves in each depositional environment undergo different amounts of transport and preservational filtering^52,53^. All three forests show distinct differences in plant species composition among depositional environments (intra-forest variability), yet reassuringly, in the temperate environments of HF and SERC, local communities are more similar to others from their own forest’s depositional environment rather than across forests (Fig. 7B). For example, HF swamp quarries are more similar to each other than SERC swamp quarries.

Depositional environment does not appear to affect FFG distributions, either within a forest or when across all quarries. This result is particularly reassuring, as many paleontological studies have examined temporal patterns in damage composition at the FFG-scale^54^. DT variability exists within each forest and the depositional environment, and this is apparent when analyzing degree (Figs. 8, 9 and 10). Degree (i.e., how many plants a DT interacts with) indicates the level of occurrence generalization. DTs group into clear communities within each quarry, suggesting that each quarry may capture a specific micro-habitat. We assume that these relationships reflect competition, mutualism, life-cycle differences, and a plethora of other moving/living variables; however, these findings, along with co-occurrence and pairwise analyses (Figs. 6 and S1–S3), emphasize that core groups of DTs remain strongly consistent within a depositional environment, even with quarry-level variability (Fig. 1). What is even more interesting is that clear, core groups of DTs are visible within each depositional environment, which differ in how far leaves are typically transported from the tree to the eventual deposition in the sediment. Even the dynamic river depositional environment, where water velocities are highest and leaf transport is expect to be greatest, displays this. This shows that fossil depositional environments are capturing the dominant DTs, even with a low resolution of data. This is an important finding which further supports that paleontological data is (1) capturing the dominant insect herbivore communities within depositional environments and (2) may provide a new framework for characterizing micro-habitats within the fossil record.

A long sought-after question within paleontology is how well leaf compression assemblages capture events within a single or few growing seasons, such as an insect outbreak. Our experimental design provides an opportunity to investigate if an event such as an outbreak could be detected, as questioned within this study. HF has been experiencing oscillating outbreak events^55^ of *Lymantria dispar* (newly renamed “spongy moths”) caterpillars for several years. *L. dispar* prefers to feed on red oak (*Quercus rubra*) leaves, although they are not specialist feeders and will consume other plant species^56^, including red maple (*Acer rubrum*), birch (*Betula* sp.), and American beech (*Fagus grandifolia*), all abundant within HF. Young caterpillars without fully mature mandibles often feed on the surfaces of leaves causing surficial damage such as DT333, a common DT at HF (Fig. 6A). As the mandible matures, generalized hole feeding is likely the dominant feeding pattern of the caterpillars^57^. Thus, the many positively occurring DTs at HF (Fig. 6) could physiologically be made by *L. dispar* caterpillars, at various life-cycle stages. Although low specialization is generally expected within temperate ecosystems^27,58^ and plant-host preference is rarely detected for generalist DTs (i.e., those that can be made by a large number of species), insect feeding preference was detected at HF via bipartite networks, specifically the H2’ metric (Fig. 5). We inadvertently found that an ecosystem experiencing an outbreak on a few plant species can be detected by collecting DT data and utilizing multiple bipartite network metrics for measuring specialization. This is exciting as we may now be able to investigate insect outbreak events within the fossil record, possibly linking patterns to climatic events.

In summary, insect herbivory varies across and within landscapes, highlighting the importance of looking at multiple metrics of insect herbivory to understand forested landscapes. This is particularly important as insect herbivory influences overall forest health by altering photosynthetic capacity^59^ and indirectly altering carbon sequestration^60–62^. The fossil record provides an opportunity to investigate past forest health using the approaches presented here. Additionally, connecting past and present insect outbreaks could hold important information regarding forest rebound, an important topic for future forests as human activities are hypothesized to influence insect herbivory^15^.

## Methods

All methods were applied to wild plants, specifically the collection of leaf material, with the permission of the Harvard Forest, Smithsonian Environmental Research Center, the Organization of Tropical Research La Selva, and in compliance with all relevant institutional, national, and international guidelines and legislation. Leaves from La Selva were exported from Costa Rica with the explicit permission of the Costa Rican government under the United States Department of Agriculture (USDA) permit number PCIP-19-00017.

### Site description

Leaves were collected from temperate Harvard Forest, USA (HF), coastal temperate Smithsonian Environmental Research Center, USA (SERC), and wet-tropical La Selva, Costa Rica (LS; Fig. 1; Table S5). HF is in Petersham, Massachusetts, and is classified as a temperate forest dominated by hemlock (*Tsuga canadensis*), various birch sp. (*Betula* sp.), red maple (*Acer rubrum*), red oak (*Quercus rubra*), and American beech (*Fagus grandifolia*). This forest experiences freeze/thaw dynamics and is located at an elevation of $\sim$1200 ft above sea level. Four depositional environments were sampled, including a low-transport swamp, mid-transport small tributary, high-transport river, and an upland location. SERC, in Edgewater, Maryland, is dominated by American beech (*Fagus grandifolia*), dogwood (*Cornus florida*), white and red oak (*Quercus alba and Quercus rubra*), sweetgum (*Liquidambar styraciflua*), tulip poplar (*Liriodendron tulipifera*), and hickory (*Carya alba*). This coastal temperate ecosystem is a combination of fresh and brackish water as the Chesapeake Bay bounds one side of the research station and creates distinct edges to the forest. Three depositional environments were targeted: low-transport swamp, mid-transport small tributary, and high-transport freshwater spring which drains into the Chesapeake Bay. LS is a low-land wet-tropical ecosystem and home to over 500 tree species, with variability in species distribution across the landscape (per comms Orlando Vargas Ramírez)^63,64^. Dominant tree species within LS are *Catilla elastica*, *Ficus insipida*, *Luehea seemannii*, *Terminalia oblonga*, and *Zygia longifolia*^65^. The station is 1600 hectares and is located within a lowland wet-tropical rainforest bound by the Rio Sarapiqui and Rio Puerto Viejo. Sites sampled were chosen to encompass various habitats and depositional environments, including a low-transport swamp within an old-growth forest, a mid-transport tributary river within an ecological reserve (undisturbed), and a high-transport dynamic river within a secondary growth forest.

During the winter (dry season) of 2019, leaf samples were collected from LS, while leaf samples from HF and SERC were collected during the summer of 2019. All depositional environments within each forest type were sampled with three replicate quarries, approximately 100 m apart, to capture lateral variability, except for the upland location which was only sampled within HF (Fig. 1). Productive leaf layers (i.e., abundant leaf material deposited within sediment layers) were identified for each depositional environment and sampled (Fig. 2). If fine-sediment accumulation was low or absent, overbank deposits were sampled using a 50m transect with random sampling occurring every 10m (Fig. 2D,F). Collections were further randomly subsampled to 400 leaves. This method was used at HF, except for two fluvial quarries which had enough fine-sediment to sample from (Fig. 2G), and within all swamp depositional environments. Each quarry consists of $\sim$400 leaves, with $\sim$1200 leaves per depositional environment, and $\sim$3600 leaves per forest. A total of 10,941 leaves were analyzed from the three forests. Leaves collected from within the sediment represent multiple growing seasons and therefore are not analyzed at the annual scale. This does not allow for the comparison of intra- and/or inter-annual variation that may occur within the forest. However, future research will work to address this knowledge gap. Leaves were then rinsed and cleaned of sediment and debris (Fig. 2H) prior to pressing to allow for optimal insect herbivore damage identification. Leaves were then dried at 60–70 °C for 48–72 h. Each leaf was identified to species when possible or sorted into a morphospecies (i.e., a designation based on distinct leaf morphology^66^) and transported to the University of Wyoming for further analysis. Leaves collected from La Selva were identified by Orlando Vargas Ramírez , all other leaves were identified by Lauren Azevedo-Schmidt. Voucher specimens are housed with the Rocky Mountain Herbarium at the University of Wyoming (Accession numbers 1052760 - 1052900). Lastly, ^14^C dating was used to date the approximate age of bulk leaf material from each depositional setting within the three forest types. UC Irvine’s KCCAMS facility performed the analyses. Ages were calibrated using NHZone 2 or NHZone 1 and the F^14^C, or fraction of modern ^14^C and calculated uncertainty^67^. Modern fractions measured range from 1.0094 to 1.0307, with minimum and maximum values occurring within the SERC swamp and small tributary, respectively. All leaves exhibit excess ^14^C from atmospheric thermonuclear weapons testing, giving an approximate age of mid-20th century or later (1955–present)^15^.

### Herbivory metrics

Plant–insect interactions were recorded within morphologically distinct feeding patterns or damage types (DTs) preserved on leaf tissue (here, we consider only the subset of DTs that occur on leaves and represent insect feeding damage). DTs are classified by their size, shape, extent, and/or location of the herbivory damage and assigned a damage type number, following Labandeira et al. (2007)^33^. A single pattern can be made by one to multiple insect species, and moreover, a single insect may be capable of creating multiple patterns of damage^7,33^, making it rare to identify specific culprits and quantify insect species diversity^68^. However, previous work has shown a positive relationship between the diversity of leaf-chewing insect herbivores and insect chewing DTs^35^. DTs are grouped into functional feeding groups (FFGs), which are similar to insect feeding guilds^33^. Analyzing an ecosystem at the DT-level allows for inferences into changes in insect species composition, while FFGs can be used to monitor broad changes among insect feeding guilds. The FFGs considered in this study are hole, margin, surface, skeletonization, piercing and sucking, gall, and mine damage. Lastly, leaf chewing damage is only quantified when thickened tissue around the damage is present to distinguish damage that occurred while the leaf was still attached to the tree (causing the thickened tissue, as the plant responds to herbivory), from damage that occurred after abscission.

Here, we quantified insect feeding damage at two resolutions: DT and FFG. Each individual leaf (n = 10,941) was observed using a dissecting scope and DT occurrence and abundance were recorded. Occurrence data is recorded as presence/absence (0 or 1) while abundance data refers to the number of incidents for each individual DT. For example, if three circular, medium holes (DT2) were present on a single leaf, the abundance is recorded as 3, while the occurrence is recorded as 1. Damage frequency, the percent of leaves with any damage, was quantified for each quarry, which can then be lumped together to obtain values at the depositional environment and forest levels. Frequencies were calculated for total, specialized, hole, margin, surface, skeletonization, piercing and sucking, gall, and mine damage. Diversity, the number of DTs in a sample, was also quantified for total, specialized, mine, and gall damage. Diversity values were standardized to 300 leaves in order to account for uneven sampling efforts, using methodologies outlined in Gunkel and Wappler (2015)^69^. Two quarries with the tributary environment at LS were omitted from further analyses due to low sample size.

### Analyses

We calculated insect herbivory frequency and diversity along with plant diversity metrics (Shannon’s diversity and Pielou’s J, a measure of evenness) at each quarry. Rosner tests (EnvStats R package; rosnerTest^70^) were used to identify and remove statistical outliers and Tukey tests were used to analyze variance in mean values across forests (agricolae R package; TukeyHSD^71^).

Random mixed effects linear models (lme4 R package; lmer^72^) were used to quantify the effects of latitude and biotic (plant diversity and evenness) variables on insect herbivory metrics. The model was run for 13 different herbivory metrics: frequencies of total, specialized, gall, mine, hole feeding, margin feeding, skeletonization, piercing and sucking, and surface feeding damage, along with diversities of total, specialized, gall, and mine damage (Tables S2 and S3). Latitude was included to encompass broad scale variability in abiotic (e.g. temperature, precipitation, and elevation) factors. Plant diversity (Shannon index) and plant species evenness (Pielou’s J) were used to understand if plant community composition predicted insect herbivory. Frequencies of FFGs were logit transformed ($logit = log[p/(1-p)]$, where p is the proportion or frequency of damage), prior to lmer analysis. Analyses were conducted at the quarry-level with forest and site included as nested random effects, accounting for the hierarchical experimental design. P-values were calculated using the Kenward-Rogers approximation, based on degrees of freedom^73^ (Tables S2 and S3). Strong predictor variables are defined here as those that notably influence the slope estimate (values $\ge$0.50) and had significant p-values.

To examine whether pairs of DTs co-occur at the scale of a leaf, we used the presence/absence of DTs at a leaf-level within each forest to calculate pair-wise strength of co-occurrence among DTs, using a metric-free, distribution-free, and randomization-free model based on a hypergeometric approach of probabilistic occurrences^74,75^. We built upon already available functionality of the ’Cooccur’ package in R and^75^, compared the observed co-occurrence to the expected co-occurrence (defined as the product of the occurrence probability of two species and sampling site count). This technique estimates if observed co-occurrences are significantly greater than expected (positive association), significantly less than expected (negative association), or not significantly different and within limits of the expected (random association; for details of implementation and review/comparison with previous co-occurrence tools^75^).

Ecological network metrics were used to quantitatively compare properties from network representations^76^. These metrics measure different aspects of plant–insect interactions, including generalized and specialized occurrence and feeding. Abundance was taken into account for each DT. In this work, we wanted to discern the differences in how plant taxa were affected at different sites. Weighted bipartite networks were used to analyze patterns in plant–insect associations at a quarry-level (for detailed methodology, see Swain et al. 2021^76^). These constructed networks had two node classes (as is the case with all bipartite networks): plant taxa and herbivory DTs^33^. An edge is present only between nodes of dissimilar node classes (i.e., a given DT and a plant taxon) and represents the occurrence of the said DT on the plant taxon. The width/weight of the network edge represents the abundance value of that interaction at the quarry-level. To standardize the comparisons and account for differences in sampling effort, we re-sampled (bootstrap) each quarry to 300 leaves (without replacement)^76^, and used those data to construct a network. This process was repeated 500 times for each quarry for statistical purposes. In each of the 500 networks at the quarry-level, we calculated these metrics at two scales: at the whole-network level (each quarry) or at node-level (DTs at each quarry)^77^. In specific we wanted to investigate patterns in proportion of interactions realized (connectance), diversity of DTs on a given plant taxon (partner diversity for plants), the secondary effect of extermination of plants on DTs (robustness), overall specialization and preference (H2’), and nested structure of interactions (nestedness)^76,78^. The proportion of realized interactions compared to the total number of possible interactions, or connectance, shows how connected DTs and plant species are, with lower connectance indicating more specialization compared to higher connectance values. High partner diversity for host-plants indicates higher diversity of plant species, which can then interact with insect herbivores, across a given landscape. An elevated robustness metric shows a more consistent assemblage of plant-DT associations with more redundancies and often with more generalized feeding behavior^79–81^. H2’, which ranges between 0 (no specialization) and 1 (complete specialization), measures how selective plant taxa or DTs are as compared to a random community (based on the same network elements as the empirical network of interest).^82^ Nestedness, as the name suggests, is a measure of how nested interactions in a network are. In a given distribution of interactions where DTs with low abundance interactions are numerous and the number of DTs with high abundance interactions are few, it can also quantify specialization vs. generalist. Interactions with lower values indicate more specialized interactions and higher values show more generalized interactions. The degree was also analyzed but only at the node or quarry scale.

We used non-metric multidimensional scaling (NMDS; vegan R package: metaMDS^83^) analyses to investigate inter- and intra-forest variability in FFG distributions and plant communities. NMDS is an analysis that produces ordinations based on a dissimilarity matrix, with more distance equating to greater difference^84^. Analyses were constructed at the quarry-level (highest resolution of our data; Fig. 1), based on the number of leaves with each FFG and belonging to each plant species. Occurrences of FFGs and plant species were then converted to proportions (between 0 and 1) for each quarry. Points in the resulting ordination were color-coded by forest with each depositional environment corresponding to a specific shape to show how herbivory and plant community composition varies spatially at the regional (inter-forest) or landscape (intra-forest) scale. Additionally, ANOSIM (analysis of similarity) was used to test if differences existed across different scales (vegan r package: anosim^83^). ANOSIM R values which are closer to 1.0 show greater similarities within groups, here, either forest or depositional environment, than among groups^85^. Inter-forest variability was tested using forest (HF, SERC, LS) and depositional environment (swamp, fluvial, tributary) as the *a priori* groups. Intra-forest variability was tested by comparing depositional environments within a forest such that the swamp, fluvial, and tributary environments within a forest were only compared to each other and not the other forests. La Selva plant communities were not directly compared to HF or SERC using NMDS/ANOSIM as they had zero plant species in common.

## Supplementary Information

Supplementary Information.

## Data Availability

The datasets generated and/or analysed during the current study are available in the Github repository, https://github.com/lazevedoschmidt/modern.landscape.herbivory

## References

[1] Currano ED, Azevedo-Schmidt L, Maccracken S, Swain A (2021). Scars on fossil leaves: An exploration of ecological patterns in plant-insect herbivore associations during the Age of Angiosperms. Palaeogeogr. Palaeoclimatol. Palaeoecol..

[2] Cariglino, B., Moisan, P. & Lara, M. B. The fossil record of plant-insect interactions and associated entomofaunas in Permian and Triassic floras from southwestern Gondwana: A review and future prospects. 10.1016/j.jsames.2021.103512 (2021).

[3] Labandeira C (2007). The origin of herbivory on land: Initial patterns of plant tissue consumption by arthropods. Insect Sci..

[4] Currano ED (2008). Sharply increased insect herbivory during the Paleocene-Eocene Thermal Maximum. Proc. Natl. Acad. Sci. USA.

[5] Azevedo-Schmidt LE, Dunn RE, Mercer J, Dechesne M, Currano ED (2019). Plant and insect herbivore community variation across the Paleocene-Eocene boundary in the Hanna Basin, southeastern Wyoming. PeerJ.

[6] Wappler T, Kustatscher E, Dellantonio E (2015). Plant-insect interactions from Middle Triassic (late Ladinian) of Monte Agnello (Dolomites, N-Italy)-initial pattern and response to abiotic environmental perturbations. PeerJ.

[7] Wilf, P. & Labandeira, C. Response of plant-insect associations to Paleocene-Eocene Warming. *Science* **284**. 10.1126/science.284.5423.2153 (1999). 10.1126/science.284.5423.2153 10381875

[8] Donovan MP, Iglesias A, Wilf P, Labandeira CC, Cuneo NR (2016). Rapid recovery of Patagonian plant-insect associations after the end-Cretaceous extinction. Nat. Ecol. Evol..

[9] Wappler T, Currano ED, Wilf P, Rust J, Labandeira CC (2009). No post-Cretaceous ecosystem depression in European forests? Rich insect-feeding damage on diverse middle Palaeocene plants, Menat, France. Proc. Biol. Sci..

[10] Wilf P, Labandeira CC, Johnson KR, Ellis B (2006). Decoupled plant and insect diversity after the end-Cretaceous extinction. Science.

[11] Labandeira CC, Johnson KR, Wilf P (2002). Impact of the terminal Cretaceous event on plant-insect associations. Proc. Natl. Acad. Sci. U S A.

[12] Azevedo-Schmidt, L. *et al.* Local differences in paleohydrology have stronger influence on plant biomarkers than regional climate change across two Paleogene Laramide Basins, Wyoming, USA. *Palaeogeogr. Palaeoclimatol. Palaeoecol.* **596**. 10.1016/j.palaeo.2022.110977 (2022).

[13] Adams JM, Ahn S, Ainuddin N, Lee M-L (2011). A further test of a palaeoecological thermometer: Tropical rainforests have more herbivore damage diversity than temperate forests. Rev. Palaeobot. Palynol..

[14] Meineke, E. K., Classen, A. T., Sanders, N. J., Jonathan Davies, T. & Iler, A. Herbarium specimens reveal increasing herbivory over the past century. *J. Ecol.* **107**, 105–117, (2018). 10.1111/1365-2745.13057

[15] Azevedo-Schmidt, L., Meineke, E. K. & Currano, E. D. Insect herbivory within modern forests is greater than fossil localities. *PNAS* **30**, 10.1073/pnas (2022). 10.1073/pnas.2202852119 PMC9586316 36215482

[16] Johnson, K. R. & Ellis, B. A tropical rainforest in Colorado 1.4 Million years after the Cretaceous-Tertiary Boundary. *Science* **296**. 10.1126/science.1072102 (2002). 10.1126/science.1072102 12089439

[17] Donovan MP, Wilf P, Labandeira CC, Johnson KR, Peppe DJ (2014). Novel insect leaf-mining after the end-Cretaceous extinction and the demise of cretaceous leaf miners, Great Plains, USA. PLoS ONE.

[18] Currano ED (2009). Patchiness and long-term change in early Eocene insect feeding damage. Paleobiology.

[19] Currano ED, Jacobs BF, Pan AD, Tabor NJ (2011). Inferring ecological disturbance in the fossil record: A case study from the late Oligocene of Ethiopia. Palaeogeogr. Palaeoclimatol. Palaeoecol..

[20] Holden, A. R., Koch, J. B., Griswold, T., Erwin, D. M. & Hall, J. Leafcutter bee nests and pupae from the Rancho La Brea Tar Pits of southern California: Implications for understanding the paleoenvironment of the Late Pleistocene. *PLoS ONE* **9**. 10.1371/journal.pone.0094724 (2014). 10.1371/journal.pone.0094724 PMC3981822 24718701

[21] Navarro, L., Harvey, A. E., Ali, A., Bergeron, Y. & Morin, H. A Holocene landscape dynamic multiproxy reconstruction: How do interactions between fire and insect outbreaks shape an ecosystem over long time scales? *PLoS ONE* **13**. 10.1371/journal.pone.0204316 (2018). 10.1371/journal.pone.0204316 PMC6168141 30278052

[22] Milbury KJ, Cwynar LC, Edwards S (2019). Distinguishing eastern North American forest moth pests by wing-scale ultrastructure: Potential applications in paleoecology. Facets.

[23] Girona, M. M., Navarro, L. & Morin, H. A secret hidden in the sediments: Lepidoptera scales. *Front. Ecol. Evol.* **6**, 10.3389/fevo.2018.00002 (2018).

[24] Fahrig, L. Effects of habitat fragmentation on biodiversity. 10.1146/annurev.ecolsys.34.011802.132419 (2003).

[25] Crist TO, Pradhan-Devare SV, Summerville KS (2006). Spatial variation in insect community and species responses to habitat loss and plant community composition. Oecologia.

[26] Burkman CE, Gardiner MM (2014). Urban greenspace composition and landscape context influence natural enemy community composition and function. Biol. Control.

[27] Coley PD, Barone JA, Barone C (1996). Herbivory and plant defenses in tropical forests. Annu. Rev. Ecol. Syst..

[28] Adams JM, Zhang Y, Basri M, Shukor N (2009). Do tropical forest leaves suffer more insect herbivory? A comparison of tropical versus temperate herbivory, estimated from leaf litter. Ecol. Res..

[29] Lim JY, Fine PV, Mittelbach GG (2015). Assessing the latitudinal gradient in herbivory. Glob. Ecol. Biogeogr..

[30] Andrew NR, Roberts IR, Hill SJ (2012). Insect herbivory along environmental gradients. Open J. Ecol..

[31] Moles AT, Bonser SP, Poore AG, Wallis IR, Foley WJ (2011). Assessing the evidence for latitudinal gradients in plant defence and herbivory. Funct. Ecol..

[32] Moles AT (2011). Putting plant resistance traits on the map: A test of the idea that plants are better defended at lower latitudes. New Phytol..

[33] Labandeira, C. C., Wilf, P., Johnson, K. R. & Marsh, F. Guide to insect (and other) damage types on compressed plant fossils. *Smithsonian Institution, National Museum of Natural History, Department of Paleobiology* **3.0** (2007).

[34] Meineke EK, Davis CC, Davies TJ (2018). The unrealized potential of herbaria for global change biology. Ecol. Monogr..

[35] Carvalho MR (2014). Insect leaf-chewing damage tracks herbivore richness in modern and ancient forests. PLoS ONE.

[36] Soh WK (2017). Palaeo leaf economics reveal a shift in ecosystem function associated with the end-Triassic mass extinction event. Nat Plants.

[37] Gaston KJ (1991). The magnitude of global insect species richness. Conserv. Biol..

[38] Godfray HCJ, Lewis OT, Memmott J (1999). Studying insect diversity in the tropics. Phil Trans. R. Soc. Lond. B..

[39] Woodward, F. I. Ecophysiological Controls of Conifer Distributions. In Mooney, H. A. (ed.) *Ecophysiology of Coniferous Forests*, 79–94. 10.1016/B978-0-08-092593-6.50009-8 (Stanford University, Stanford, California, 1995).

[40] Givnish, T. Leaf and canopy adaptations in tropical forests. *Physiological ecology of plants of the we tropics* 51–84. 10.1007/978-94-009-7299-5_6 (1984).

[41] Strauss-Debenedetti S, Bazzaz FA (1991). Plasticity and acclimation to light in tropical Moraceae of different sucessional positions. Oecologia.

[42] Archibald SB, Bossert WH, Greenwood DR, Farrell BD (2016). Seasonality, the latitudinal gradient of diversity, and Eocene insects. Paleobiology.

[43] Sinclair, R. J. & Hughes, L. Leaf miners: The hidden herbivores. *Austral. Ecol.* **35**, 300–313. 10.1111/j.1442-9993.2009.02039.x (2010).

[44] De Souza Mendonca, M. & Jr.,. Galling insect diversity patterns: the resource synchronization hypothesis. *Oikos* **95**(1), 171–176. 10.1034/j.1600-0706.2001.950120.x (2001).

[45] Danks HV (2006). Key themes in the study of seasonal adaptations in insects II. Life-Cycle Patt..

[46] González E, Salvo A, Valladares G (2015). Arthropods on plants in a fragmented Neotropical dry forest: A functional analysis of area loss and edge effects. Insect Sci..

[47] Lincoln DE, Fajer ED, Johnson RH (1993). Plant-Insect Herbivore Interactions in Elevated CO2 Environments. TREE.

[48] Turner, M. G. Landscape ecology: The effect of pattern on process. *Annu. Rev. Ecol. Syst.* **20**, 171–197 (1989).

[49] Diekötter T, Billeter R, Crist TO (2008). Effects of landscape connectivity on the spatial distribution of insect diversity in agricultural mosaic landscapes. Basic Appl. Ecol..

[50] Soberon J (2007). Grinnellian and Eltonian niches and geographic distributions of species. Ecol. Lett..

[51] Heinen R, van der Sluijs M, Biere A, Harvey JA, Bezemer TM (2018). Plant community composition but not plant traits determine the outcome of soil legacy effects on plants and insects. J. Ecol..

[52] Burnham RJ, Wing S, Parker GG (1992). The reflection of deciduous forest communities in leaf litter: Implications for autochthonous litter assemblages from the fossil record. Paleobiology.

[53] Burnham RJ (1993). Reconstructing richness in the plant fossil record. Palaios.

[54] Currano ED, Labandeira C, Wilf P (2010). Fossil insect folivory tracks paleotemperature for six million years. Ecol. Monogr..

[55] Henry, B., Spurr, S. H. & Littlefield, E. *Forest site conditions and the gypsy moth*, vol. 22 (1947).

[56] Traw MB, Bazzaz FA, Lindroth RL (1996). Decline in gypsy moth (Lymantria dispar) performance in an elevated CO 2 atmosphere depends upon host plant species. Oecologia.

[57] Bernays EA, Jarzembowski EA, Malcolm SB (1991). Evolution of Insect Morphology in Relation to Plants [and Discussion]. Philos. Trans. Biol. Sci..

[58] Paine RT (1966). Food Web Complexity and Species Diversity. Ame. Soc. Nat..

[59] Zangerl AR (2002). Impact of folivory on photosynthesis is greater than the sum of its holes. Proc. Natl. Acad. Sci. U S A.

[60] Luyssaert S (2008). Old-growth forests as global carbon sinks. Nature.

[61] Whitehead D (2011). Forests as carbon sinks-benefits and consequences. Tree Physiol..

[62] Pugh TA (2019). Role of forest regrowth in global carbon sink dynamics. Proc. Natl. Acad. Sci. U.S.A..

[63] Newstrom LE, Frankie GW, Baker HG (1994). A New Classification for Plant Phenology Based on Flowering Patterns in Lowland Tropical Rain Forest Trees at La Selva. Costa Rica. Biotropica.

[64] Clark, D. B. & Clark, D. A. Landscape-scale variation in forest structure and biomass in a tropical rain forest. *For. Ecol. Manage.* **137.1-3**, 185–198. 10.1016/S0378-1127(99)00327-8 (2000).

[65] Zhang J, Rivard B, Sánchez-Azofeifa A, Castro-Esau K (2006). Intra- and inter-class spectral variability of tropical tree species at La Selva, Costa Rica: Implications for species identification using HYDICE imagery. Remote Sens. Environ..

[66] Ellis, B. *et al.**Manual of Leaf Architecture* (Cornell University Press, 2009).

[67] Reimer, P. J., Brown, T. A. & Reimer, R. W. Discussion: Reporting and calibration of post-bomb 14C data. *Radiocarbon* **46**(3), 12991304. 10.1017/S0033822200033154 (2004).

[68] Maccracken, S. A., Miller, I. M., Johnson, K. R., Sertich, J. M. & Labandeira, C. C. Insect herbivory on Catula gettyi gen. et sp. nov. (Lauraceae) from the Kaiparowits Formation (Late Cretaceous, Utah, USA). *PLoS ONE* **17**. 10.1371/journal.pone.0261397 (2022). 10.1371/journal.pone.0261397 PMC8782542 35061696

[69] Gunkel S, Wappler T (2015). Plant-insect interactions in the upper Oligocene of Enspel (Westerwald, Germany), including an extended mathematical framework for rarefaction. Palaeobiodivers. Palaeoenviron..

[70] Millard SP (2013). EnvStats: An R Package for Environmental Statistics.

[71] de Mendiburu, F. agricolae tutorial (Version 1.3-5). *Universidad Nactional Agraria: La Molina, Puru* (2021).

[72] Bates, D., Mächler, M., Bolker, B. M. & Walker, S. C. Fitting linear mixed-effects models using lme4. *J. Stat. Softw.* **67**. 10.18637/jss.v067.i01 (2015).

[73] Kenward MG, Roger JH (1997). Small sample inference for fixed effects from restricted maximum likelihood. Biometrics.

[74] Veech JA (2013). A probabilistic model for analysing species co-occurrence. Glob. Ecol. Biogeogr..

[75] Griffith, D. M., Veech, J. A. & Marsh, C. J. Cooccur: Probabilistic species co-occurrence analysis in R. *J. Stat. Softw.* **69**. 10.18637/jss.v069.c02 (2016).

[76] Swain, A., Maccracken, S. A., Fagan, W. F. & Labandeira, C. C. Understanding the ecology of host plant-insect herbivore interactions in the fossil record through bipartite networks. *Paleobiology*. 10.1017/pab.2021.20 (2021).

[77] Dormann, C. F., Fründ, J., Blüthgen, N. & Gruber, B. Indices, Graphs and Null Models: Analyzing Bipartite Ecological Networks. *Open Ecol. J.* **2**, 7–24. 10.2174/1874213000902010007 (2009).

[78] Swain, A. *et al.* Sampling bias and the robustness of ecological metrics for fossil plant-damage-type association networks. *Ecology (in Review)*. 10.1002/ecy.3922 (2022). 10.1002/ecy.3922 36415050

[79] Memmott, J., Waser, N. M. & Price, M. V. Tolerance of pollination networks to species extinctions. *Proc. R. Soc. B Biol. Sci.* **271**, 2605–2611. 10.1098/rspb.2004.2909 (2004). 10.1098/rspb.2004.2909 PMC1691904 15615687

[80] Zitnik M, Sosič R, Feldman MW, Leskovec J (2019). Evolution of resilience in protein interactomes across the tree of life. Proc. Natl. Acad. Sci. U.S.A..

[81] Klein, B. *et al.* A computational exploration of resilience and evolvability of protein-protein interaction networks. *Commun. Biol.* **4**. 10.1038/s42003-021-02867-8 (2021). 10.1038/s42003-021-02867-8 PMC8639913 34857859

[82] Blüthgen, N., Menzel, F. & Blüthgen, N. Measuring specialization in species interaction networks. *BMC Ecol.* **6**. 10.1186/1472-6785-6-9 (2006). 10.1186/1472-6785-6-9 PMC1570337 16907983

[83] Oksanen, J. *et al.* Package ’vegan’ Title Community Ecology Package Version 2.5-7 (2013).

[84] Legendre P, Gallagher ED (2001). Ecologically meaningful transformations for ordination of species data. Oecologia.

[85] Anderson, M. J. & Walsh, D. C. I. PERMANOVA, ANOSIM, and the Mantel test in the face of heterogeneous dispersions: What null hypothesis are you testing? 10.1890/12-2010.1 (2013).

