# Supplementary material for: Landscape-level variability and insect herbivore outbreak captured within modern forests provides a framework for interpreting the fossil record
Source: Sci Rep. 2023 Jun 15;13:9701. doi: 10.1038/s41598-023-36763-4 (PMC10272219; doi:10.1038/s41598-023-36763-4)
Supplement: Supplementary file 1 — Supplementary Information. [file 41598_2023_36763_MOESM1_ESM.pdf]

Supplementary Figures and Tables

Forest	Metric	Diff	Lwr	Upr	P (adj)	Significance
**SERC - La Selva**	Total Damage Frequency	-4.514603	-14.302315	5.273109	0.4910915	
**Harvard - La Selva**	Total Damage Frequency	3.998286	-5.5729258	13.569497	0.5561263	
**Harvard - SERC**	Total Damage Frequency	8.512889	-0.4108534	17.436631	0.0634393	
**SERC - La Selva**	Total Damage Diversity	-4.195873	-9.419831	1.028085	0.1319985	
**Harvard - La Selva**	Total Damage Diversity	-3.2844286	-8.392835	1.823977	0.2616453	
**Harvard - SERC**	Total Damage Diversity	0.9114444	-3.85139	5.674279	0.8817299	
**SERC - La Selva**	Specialized Frequency	-19.931746	-10.85667	0.9931806	0.0639017	
**Harvard - La Selva**	Specialized Frequency	-19.331857	-39.79393	1.1302176	0.0664789	
**Harvard - SERC**	Specialized Frequency	0.5998889	-18.47798	19.677754	0.9965876	
**SERC - La Selva**	Specialized Diversity	-1.575079	-5.409591	2.2594326	0.5667503	
**Harvard - La Selva**	Specialized Diversity	-2.852857	-6.602551	0.8968368	0.1598244	
**Harvard - SERC**	Specialized Diversity	-1.277778	-4.773814	2.2182585	0.636316	
**SERC - La Selva**	Mine Frequency	-7.6593651	-14.44027	-0.8784566	0.0248417	*
**Harvard - La Selva**	Mine Frequency	-7.6061429	-14.23706	-0.9752256	0.022532	*
**Harvard - SERC**	Mine Frequency	0.05322222	-6.12913	6.235574	0.9997438	
**SERC - La Selva**	Mine Diversity	-1.6053968	-3.537491	0.3266977	0.1160474	
**Harvard - La Selva**	Mine Diversity	-2.4642857	-4.353643	-0.5749284	0.0091513	**
**Harvard - SERC**	Mine Diversity	-0.8588889	-2.620436	0.902658	0.4531348	
**SERC - La Selva**	Gall Frequency	-27.21476	-36.847864	-17.58166	0.000001	***
**Harvard - La Selva**	Gall Frequency	-13.45743	-22.87745	-4.037407	0.0043752	**
**Harvard - SERC**	Gall Frequency	13.75733	4.974554	22.540113	0.0018969	**
**SERC - La Selva**	Gall Diversity	-4.0103175	-5.301452	-2.719183	0.0000002	*****
**Harvard - La Selva**	Gall Diversity	-3.8804286	-5.143004	-2.617853	0.0000002	*****
**Harvard - SERC**	Gall Diversity	0.1298889	-1.047276	1.307054	0.9588562	
**SERC - La Selva**	Hole Feeding Frequency	-19.188571	-317826160	-6.594527	0.0024624	**
**Harvard - La Selva**	Hole Feeding Frequency	-6.979571	-19.29504	5.335897	0.347838	
**Harvard - SERC**	Hole Feeding Frequency	12.209	0.7266427	23.691357	0.0357096	*
**SERC - La Selva**	Margin Feeding Frequency	9.932857	-2.341223	22.20694	0.1278924	
**Harvard - La Selva**	Margin Feeding Frequency	19.272857	6.998777	31.54694	0.0019184	**
**Harvard - SERC**	Margin Feeding Frequency	9.34	-2.141351	20.82135	0.1254146	
**SERC - La Selva**	Skeletonized Feeding Frequency	8.517143	1.854745	15.17954	0.010646	*
**Harvard - La Selva**	Skeletonized Feeding Frequency	7.909143	1.394115	14.424171	0.0154363	*
**Harvard - SERC**	Skeletonized Feeding Frequency	-0.608	-6.682302	5.466302	0.9660057	
**SERC - La Selva**	Surface Feeding Frequency	5.6184127	-15.35454	26.59137	0.7825005	
**Harvard - La Selva**	Surface Feeding Frequency	0.6658571	-19.84318	21.1749	0.9963625	
**Harvard - SERC**	Surface Feeding Frequency	-4.9525556	-24.07421	14.1691	0.7949845	
**SERC - La Selva**	Piercing and Sucking Feeding Frequency	0.54625	0.00424502	1.08825498	0.0480378	*
**Harvard - La Selva**	Piercing and Sucking Feeding Frequency	0.05571429	-0.5040661	0.61549462	0.9654343	
**Harvard - SERC**	Piercing and Sucking Feeding Frequency	-0.4905357	-1.0325407	0.05146927	0.0802886	
**SERC - La Selva**	Shannon Diversity (Leaf)	-0.1061905	-0.6537223	0.4413413	0.878743	
**Harvard - La Selva**	Shannon Diversity (Leaf)	0.8328571	0.2974365	1.3682778	0.0020273	**
**Harvard - SERC**	Shannon Diversity (Leaf)	0.7266667	0.227466	1.2258678	0.0037175	**
**SERC - La Selva**	Pj (Evenness; Leaf)	0.06539683	-0.0946897	0.22548332	0.5701919	
**Harvard - La Selva**	Pj (Evenness; Leaf)	-0.0057143	-0.1622597	0.15083115	0.9954044	
**Harvard - SERC**	Pj (Evenness; Leaf)	-0.0711111	-0.2170666	0.07484441	0.4536534	

Supplementary Table 1. Output table for linear models testing differences across forests and herbivory metrics

	Total Damage (%)			Specialized Damage (%)			Gall Damage (%)			Mine Damage (%)		
Predictors	*Estimates*	*CI*	*p*	*Estimates*	*CI*	*p*	*Estimates*	*CI*	*p*	*Estimates*	*CI*	*p*
Latitude	0.21	-0.81 – 1.24	0.666	-0.33	-0.85 – 0.18	0.187	-0.61	-2.41 – 1.19	0.486	-0.33	-0.83 – 0.17	0.184
Plant Diversity (Shannon)	0.16	-0.80 – 1.13	0.724	0.06	-0.62 – 0.74	0.853	0.26	-0.44 – 0.96	0.448	0.22	-0.26 – 0.70	0.354
Plant Evenness	-0.17	-0.91 – 0.57	0.632	-0.15	-0.71 – 0.41	0.582	-0.32	-0.83 – 0.18	0.194	-0.05	-0.40 – 0.29	0.763
**Random Effects**												
σ^2	0.73			0.47			0.31			0.15		
τ_{00}	0.06 Facies:Forest			0.14 Facies:Forest			0.16 Facies:Forest			0.36 Facies:Forest		
	0.50 Forest			0.00 Forest			2.22 Forest			0.00 Forest		
ICC	0.43						0.88					
N	9 Facies			9 Facies			9 Facies			9 Facies		
	3 Forest			3 Forest			3 Forest			3 Forest		
Observations	26			26			26			26		
Marginal R^2 / Conditional R^2	0.019 / 0.444			0.245 / NA			0.182 / 0.906			0.586 / NA		
AIC	83.378			74.838			70.36			58.87		

	Hole Feeding Damage (%)			Margin Feeding Damage (%)			Skeletonization (%)			Piercing & Sucking (%)			Surface Feeding (%)		
Predictors	*Estimates*	*CI*	*p*	*Estimates*	*CI*	*p*	*Estimates*	*CI*	*p*	*Estimates*	*CI*	*p*	*Estimates*	*CI*	*p*
Latitude	-0.32	-0.72 – 0.09	0.119	0.37	-0.25 – 1.00	0.226	0.53	0.12 – 0.94	**0.014**	0.16	-0.08 – 0.41	0.173	0.15	-0.40 – 0.70	0.572
Plant Diversity (Shannon)	-0.18	-0.59 – 0.23	0.365	0.03	-0.65 – 0.71	0.928	0.07	-0.47 – 0.61	0.786	0.05	-0.12 – 0.22	0.538	0.24	-0.48 – 0.96	0.497
Plant Evenness	0.04	-0.26 – 0.35	0.766	-0.04	-0.57 – 0.49	0.886	0	-0.44 – 0.45	0.991	0.01	-0.10 – 0.13	0.845	-0.23	-0.82 – 0.37	0.433
**Random Effects**															
σ^2	0.12			0.38			0.29			0.02			0.51		
τ_{00}	0.12 Facies:Forest			0.05 Facies:Forest			0.10 Facies:Forest			0.11 Facies:Forest			0.18 Facies:Forest		
	0.03 Forest			0.14 Forest			0.00 Forest			0.00 Forest			0.00 Forest		
ICC	0.57			0.33											
N	9 Facies			9 Facies			9 Facies			9 Facies			9 Facies		
	3 Forest			3 Forest			3 Forest			3 Forest			3 Forest		
Observations	26			26			26			26			26		
Marginal R^2 / Conditional R^2	0.203 / 0.661			0.183 / 0.454			0.469 / NA			0.595 / NA			0.031 / NA		
AIC	49.588			69.569			64.447			15.955			77.167		

Supplementary Table 2. Summary model output of latitude and plant community composition effects on total, specialized, gall, mine, hole feeding, margin feeding, skeletonization, piercing & sucking, and surface feeding damage frequencies.

	Total Damage Diversity			Specialized Damage Diversity			Gall Damage Diversity			Mine Damage Diversity		
Predictors	*Estimates*	*CI*	*p*	*Estimates*	*CI*	*p*	*Estimates*	*CI*	*p*	*Estimates*	*CI*	*p*
Latitude	-0.53	-3.82 – 2.75	0.738	-0.74	-2.95 – 1.47	0.492	-1.98	-2.60 – -1.36	**<0.001**	-0.42	-1.49 – 0.66	0.427
Plant Diversity (Shannon)	1.9	-1.93 – 5.74	0.312	1.02	-1.61 – 3.66	0.427	-0.46	-1.34 – 0.43	0.292	1.13	-0.13 – 2.40	0.077
Plant Evenness	-1.93	-4.88 – 1.01	0.186	-0.47	-2.50 – 1.55	0.631	0.1	-0.69 – 0.89	0.792	-0.38	-1.35 – 0.59	0.418
**Random Effects**												
σ^2	11.67			5.5			1.05			1.25		
τ_{00}	6.98 $_{Facies:Forest}$			4.74 $_{Facies:Forest}$			0.00 $_{Facies:Forest}$			1.17 $_{Facies:Forest}$		
	1.42 $_{Forest}$			0.00 $_{Forest}$			0.00 $_{Forest}$			0.00 $_{Forest}$		
ICC	0.42									0.48		
N	9 $_{Facies}$			9 $_{Facies}$			9 $_{Facies}$			9 $_{Facies}$		
	3 $_{Forest}$			3 $_{Forest}$			3 $_{Forest}$			3 $_{Forest}$		
Observations	26			26			26			26		
Marginal R^2 / Conditional R^2	0.146 / 0.504			0.252 / NA			0.754 / NA			0.373 / 0.676		
AIC	148.223			132.98			88.946			100.717		

Supplementary Table 3. Summary model output of latitude and plant community composition effects on total, specialized, gall, and mine damage diversities.

	Difference			P - value		
	La Selva - Harvard Forest	SERC – Harvard Forest	SERC – La Selva	La Selva – Harvard Forest	SERC – Harvard Forest	SERC – La Selva
**connectance**	-0.11388	-0.0321318	0.18673895	**4.27E-05**	**0.00667301**	0.08472115
**nestedness**	-1.8073542	0.41969688	7.03597718	**0.00489851**	0.08402993	0.35633685
**NODF**	1.08032548	4.44275775	9.92769452	0.12178681	0.86113492	0.29451025
**weighted connectance**	0.04620507	0.05461762	0.05055464	0.87706473	0.41428071	0.7666455
**interaction evenness**	0.16732923	0.18230822	0.11967853	0.17326282	**0.04130116**	0.84868388
**Alatalo interaction evenness**	0.21151913	0.15385819	0.09912376	0.47705849	0.92773792	0.69691365
**H2’**	-0.0659827	-0.0320901	0.18183571	**0.00308965**	**0.01228921**	0.72037837
**number.of.species.HL**	8.48186288	3.84938409	0.9486071	0.2475729	0.8793051	0.12281571
**number.of.species.LL**	19.2842616	10.9189661	-3.5855888	**4.28E-08**	**0.00099574**	**0.00045048**
**mean.number.of.shared.partners.HL**	0.64976818	0.67697006	0.45397255	0.24460503	0.10355496	0.93988008
**mean.number.of.shared.partners.LL**	-1.3885332	-0.4085632	2.81511514	**0.0003856**	**0.01182226**	0.26938205
**niche.overlap.HL**	0.04379724	0.00223403	0.14427883	0.17280406	0.05380395	0.90290022
**niche.overlap.LL**	0.13913204	0.1360402	0.14030668	0.98850936	0.95956215	0.99358047
**C.score.HL**	0.19886473	0.17300727	0.10641581	0.2655579	0.39556098	0.93262581
**C.score.LL**	0.11716035	0.05919172	0.0225306	0.32360644	0.93454109	0.20628638
**robustness.HL**	-0.0381596	-0.0124264	0.08163876	**0.0006353**	**0.01185092**	0.36009831
**robustness.LL**	0.06567579	0.07434748	0.0519929	0.25160966	**0.04823121**	0.7658017
**partner.diversity.HL**	1.23522091	1.05234169	0.23682587	**3.64E-05**	**0.00019888**	0.58565783
**partner.diversity.LL**	0.67809148	0.51593584	0.21852567	0.06740169	0.3259177	0.60166208

Supplementary Table 4. Supplementary table of bipartite network-level metrics summary output.

Forest	Dep. Env	Quarry	Lat	Long
**La Selva**	Fluvial	LS1901.1	10.430317	-82.0041
**La Selva**	Fluvial	LS1901.2	10.430067	-82.004033
**La Selva**	Fluvial	LS1901.3	10.42955	-84.003833
**La Selva**	Tributary	LS1902.1	10.4291	-84.0063
**La Selva**	Tributary	LS1902.2	10.4296	-84.0062
**La Selva**	Tributary	LS1902.3	10.429817	-84.00605
**La Selva**	Swamp	LS1903.1	10.415433	-84.005067
**La Selva**	Swamp	LS1903.2	10.41385	-84.0059
**La Selva**	Swamp	LS1903.3	10.414583	-84.00545
**Harvard Forest**	Swamp	HF1901.1	42.536367	-72.177433
**Harvard Forest**	Swamp	HF1901.2	42.539317	-72.180067
**Harvard Forest**	Swamp	HF1901.3	42.538267	-72.181883
**Harvard Forest**	Tributary	HF1902.1	42.532467	-72.188183
**Harvard Forest**	Tributary	HF1902.2	42.5312	-72.188583
**Harvard Forest**	Tributary	HF1902.3	42.5336	-72.18805
**Harvard Forest**	Fluvial	HF1903.1	42.4725	-72.161733
**Harvard Forest**	Fluvial	HF1903.2	42.471283	-72.161933
**Harvard Forest**	Fluvial	HF1903.3	42.475337	-72.1612
**Harvard Forest**	Upland	HF1903.4	42.470617	-72.162383
**SERC**	Swamp	MD1901.1	38.874717	-76.552767
**SERC**	Swamp	MD1901.2	38.873	-76.553233
**SERC**	Swamp	MD1901.3	38.874667	-76.5511
**SERC**	Tributary	MD1902.1	38.887767	-76.5638
**SERC**	Tributary	MD1902.2	38.886833	-76.563267
**SERC**	Tributary	MD1902.3	38.888283	-76.559083
**SERC**	Fluvial	MD1903.1	38.87625	-76.548583
**SERC**	Fluvial	MD1903.2	38.8763	-76.546483
**SERC**	Fluvial	MD1903.3	38.878283	-76.54545

Supplementary Table 5. GPS points for each quarry sampled for this study

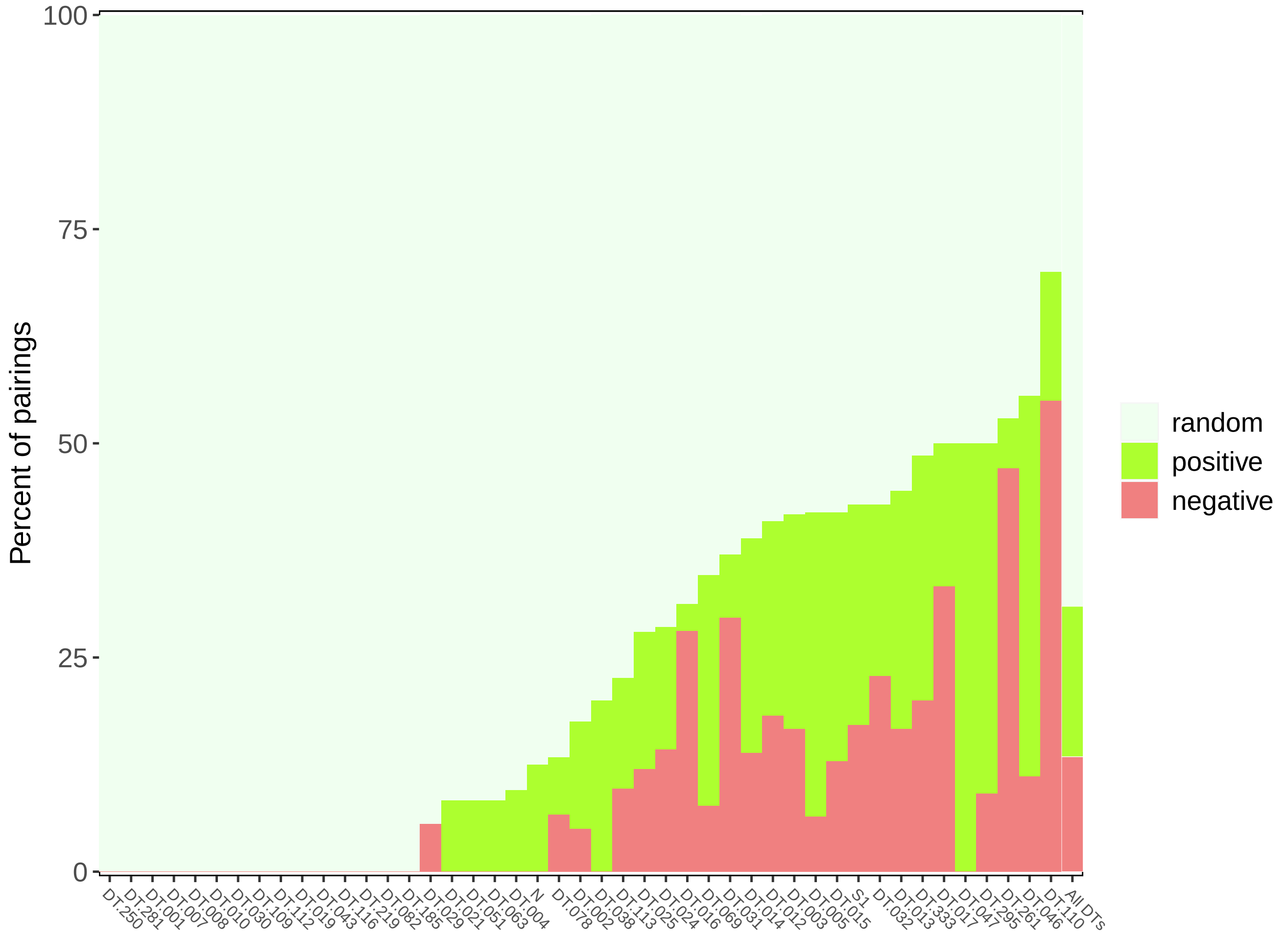

Supplementary Figure 1. Harvard Forest pair-wise comparison. Green values are positive while pink are negative while mint green is random. If the bar is one color, green or pink, that means the DT occurs 100% of the time, positively or negatively, with the other DTs. If the bar is split colors than the percentage is taken from the y-axis.

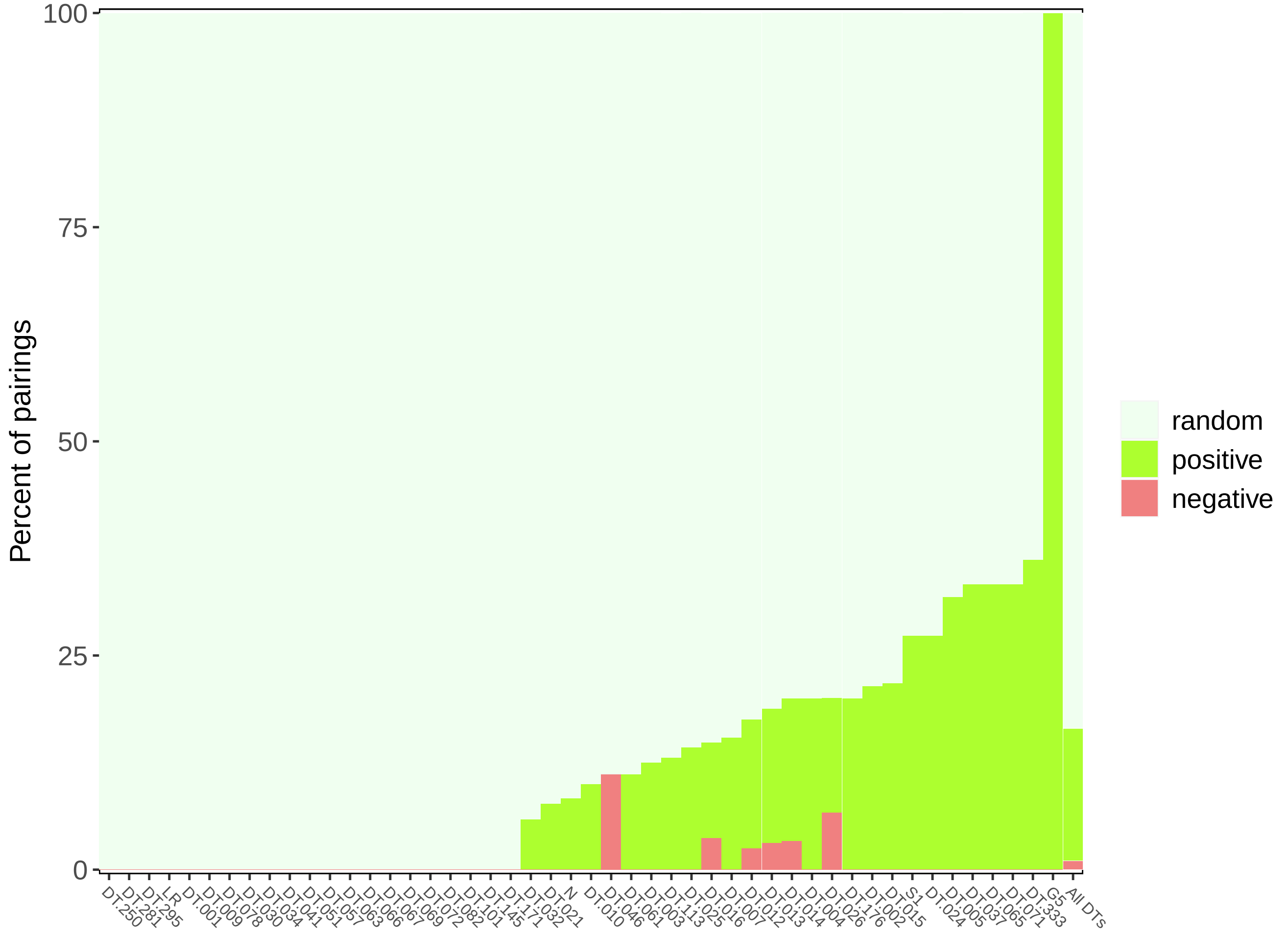

Supplementary Figure 2. SERC pair-wise comparison. Green values are positive while pink are negative while mint green is random. If the bar is one color, green or pink, that means the DT occurs 100% of the time, positively or negatively, with the other DTs. If the bar is split colors than the percentage is taken from the y-axis.

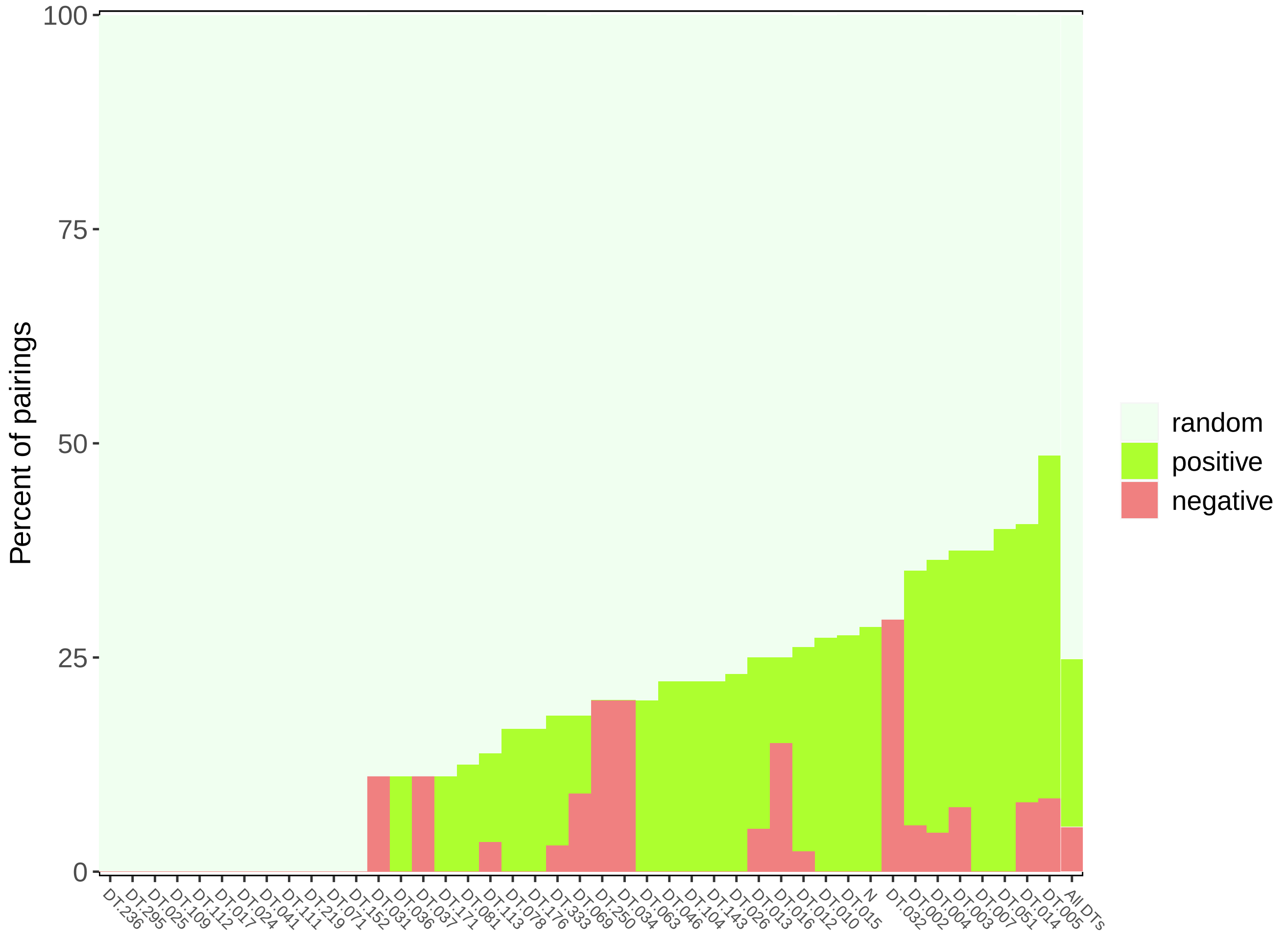

Supplementary Figure 3. La Selva pair-wise comparison. Green values are positive while pink are negative while mint green is random. If the bar is one color, green or pink, that means the DT occurs 100% of the time, positively or negatively, with the other DTs. If the bar is split colors than the percentage is taken from the y-axis.

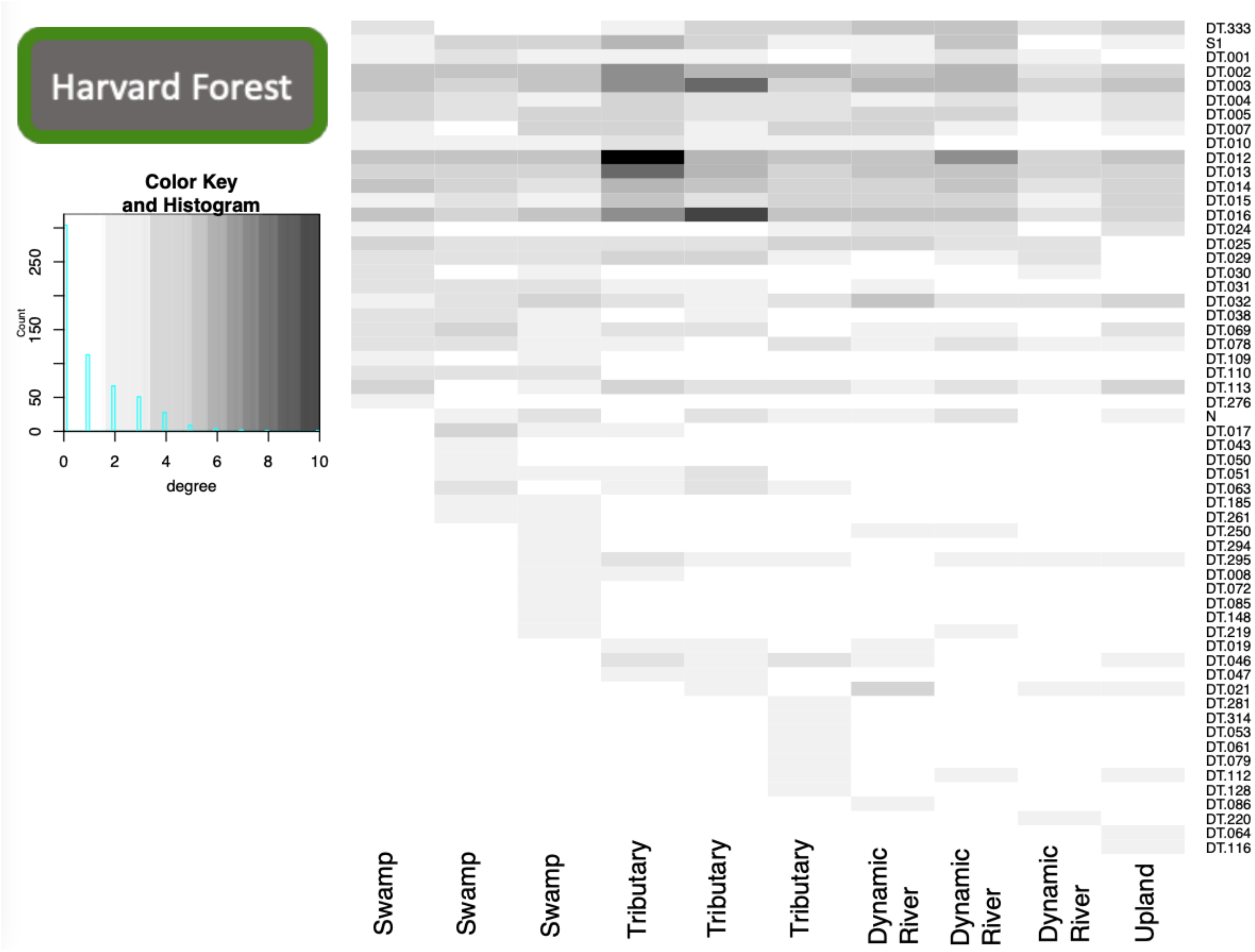

Supplementary Figure 4. Supplementary figure showing Harvard Forest bipartite network metric, degree, at the node-level with singletons removed.

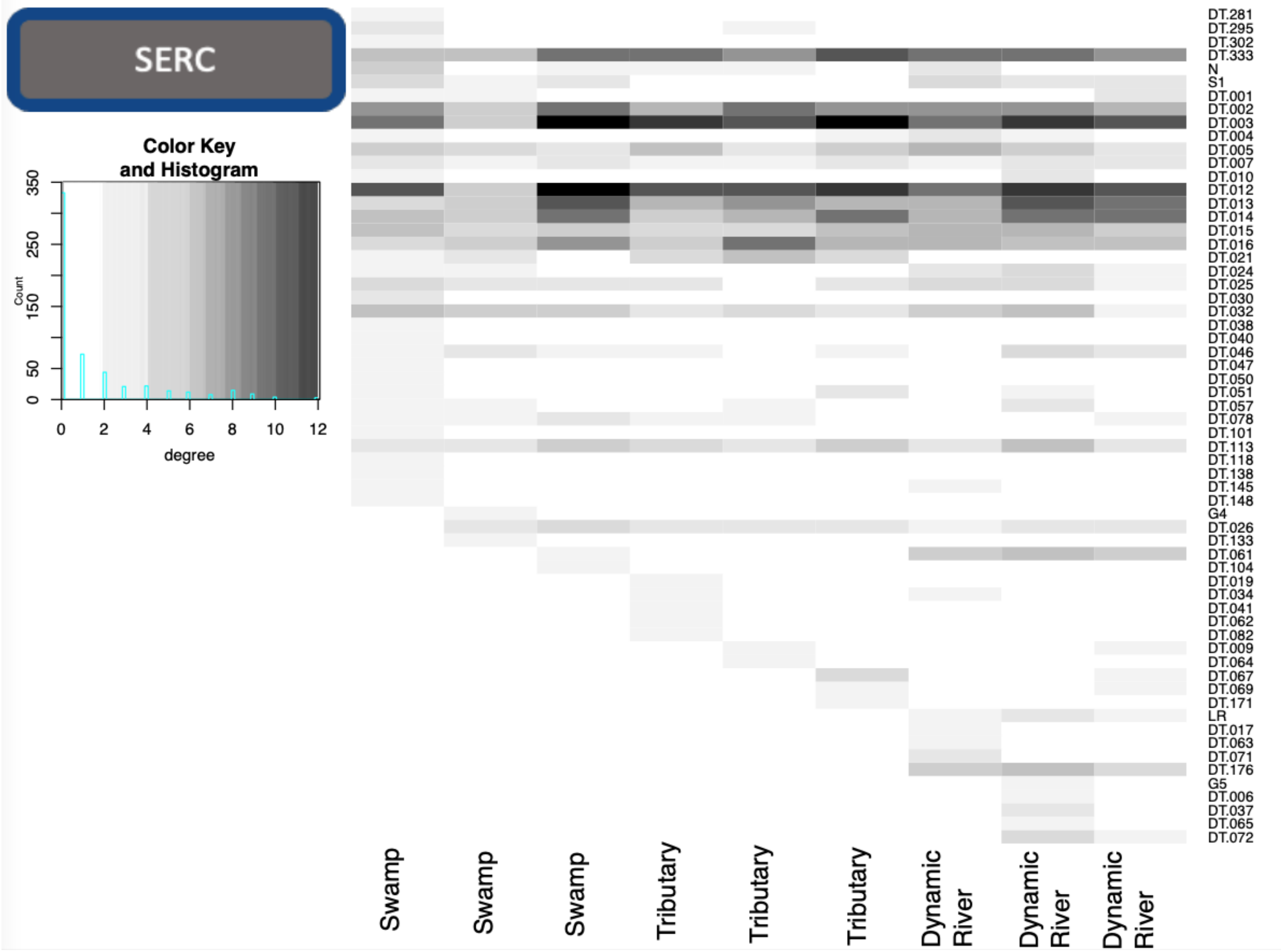

Supplementary Figure 5. Supplementary figure showing SERC bipartite network metric, degree, at the node-level with singletons removed.

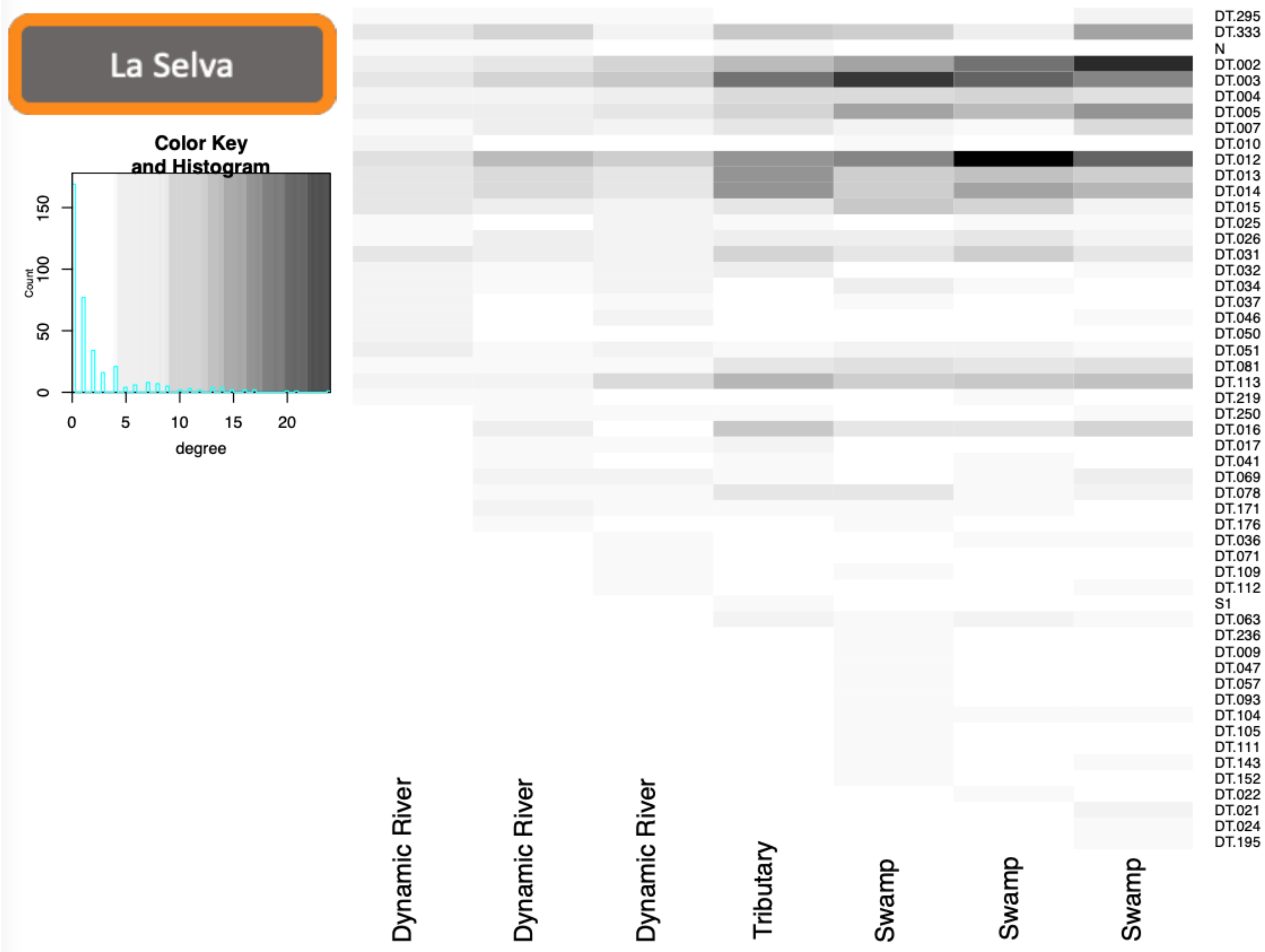

Supplementary Figure 6. Supplementary figure showing La Selva bipartite network metric, degree, at the node-level with singletons removed.
